# NOX2 Activation in COVID-19: Possible Implications for Neurodegenerative Diseases

**DOI:** 10.3390/medicina57060604

**Published:** 2021-06-11

**Authors:** Cinzia Sindona, Giovanni Schepici, Valentina Contestabile, Placido Bramanti, Emanuela Mazzon

**Affiliations:** IRCCS Centro Neurolesi “Bonino-Pulejo”, Via Provinciale Palermo, Contrada Casazza, 98124 Messina, Italy; cinzia.sindona@irccsme.it (C.S.); giovanni.schepici@irccsme.it (G.S.); valentina.contestabile@irccsme.it (V.C.); placido.bramanti@irccsme.it (P.B.)

**Keywords:** COVID-19, NOX2, neurodegenerative disease, oxidative stress, central nervous system

## Abstract

Coronavirus disease 2019 (COVID-19) is a rapidly spreading contagious infectious disease caused by the pathogen severe acute respiratory syndrome coronavirus 2 (SARS-CoV-2), that primarily affects the respiratory tract as well as the central nervous system (CNS). SARS-CoV-2 infection occurs through the interaction of the viral protein Spike with the angiotensin II receptor (ACE 2), leading to an increase of angiotensin II and activation of nicotinamide adenine dinucleotide phosphate oxidase2 (NOX2), resulting in the release of both reactive oxygen species (ROS) and inflammatory molecules. The purpose of the review is to explain that SARS-CoV-2 infection can determine neuroinflammation that induces NOX2 activation in microglia. To better understand the role of NOX2 in inflammation, an overview of its involvement in neurodegenerative diseases (NDs) such as Parkinson’s disease (PD), Alzheimer’s disease (AD), and amyotrophic lateral sclerosis (ALS) is provided. To write this manuscript, we performed a PubMed search to evaluate the possible relationship of SARS-CoV-2 infection in NOX2 activation in microglia, as well as the role of NOX2 in NDs. Several studies highlighted that NOX2 activation in microglia amplifies neuroinflammation. To date, there is no clinical treatment capable of counteracting its activation, however, NOX2 could be a promising pharmaceutical target useful for both the treatment and prevention of NDs and COVID-19 treatment.

## 1. Introduction

Coronavirus disease 2019 (COVID-19) represents a worldwide problem caused by severe acute respiratory syndrome coronavirus 2 (SARS-CoV-2) identified in December 2019 in Wuhan, China [1].

Several modes of transmission of SARS-CoV-2 are known, but the dominant one is respiratory, through aerosols. Other transmission modalities are hypothesized such as contact with contaminated surfaces or fecal-oral transmission [2]. 

Although many patients are asymptomatic or mildly symptomatic, SARS-CoV-2 infection manifests itself not only as viral pneumonia but as a very complex multi-organ disease whose pathophysiological basis is not fully known [3]. In addition to mild or moderate respiratory symptoms, severe symptoms such as dyspnea, hypoxia, and possible complications such as acute respiratory distress syndrome (ARDS) and thromboembolism were observed in SARS-CoV-2 patients. However, neurological manifestations, particularly in the central nervous system (CNS) such as headache, dizziness, impaired consciousness and motor coordination, acute cerebrovascular disease, and epilepsy have been observed in SARS-CoV-2 patients. Instead, symptoms associated with the peripheral nervous system (SNP) including anosmia and ageusia that could be considered early markers of the disease [4]. 

Recent studies have reported that SARS-CoV-2 can act also indirectly into the CNS. Indeed, it was shown that the disease is the most severe form, can manifest itself as an inflammatory syndrome due to excessive production and release of inflammatory cytokines, as well as by dysfunction of the immune response. SARS-CoV-2 patients showed elevated levels of pro-inflammatory cytokines including tumor necrosis factor-α (TNF-α), interleukin-1beta (IL-1β), interleukin-2 (IL-2), and interleukin-6 (IL-6) that activate specific transcription factors such as nuclear factor-κB (NF-κB), which by inducing the expression of pro-inflammatory genes, as well as by regulating the inflammasome can contribute to neuroinflammation [5].

SARS-CoV-2 infection can induce the activation of microglia, resident immune cells of the CNS, that play a role in both the regulation of homeostasis and inflammatory response [6]. The pathophysiological insults promoted by a viral infection or hypoxic condition determine the activation of microglia, which in turn activate the nicotinamide adenine dinucleotide phosphate (NADPH) oxidase (NOX) enzyme, responsible for the production of superoxide anion (O_2_^−^) [7,8]. 

Therefore, in response to the increase in both inflammatory cytokines, Ang II, glucose, and oxidized LDL, NOX2 is activated to generate O_2_^−^, the latter responsible for cellular senescence and aging [9,10]. 

Therefore, NOX2 activation can induce a notable increase of reactive oxygen species (ROS) which are considered responsible for neuronal oxidative damage, to favor the development and progression of neurodegenerative diseases (NDs) [11]. Several pieces of evidence show that microglial activation of NOX2 is implicated in the pathogenesis of NDs including Alzheimer’s disease (AD), Parkinson’s disease (PD), and Amyotrophic Lateral Sclerosis (ALS) [8]. 

Recently, Violi et al. investigated the role of NOX2 and its possible involvement in the pathophysiological mechanism of COVID-19. This investigation was conducted on 182 hospitalized COVID-19 patients which demonstrated a higher serum NOX2 dosage, specifically in patients who needed mechanical ventilation or with thrombosis, so suggesting a connection between NOX2 activation and clinical worsening [12].

The purpose of this review is to provide NOX2 aspects, specifically its activation following viral infections such as SARS-CoV-2. Furthermore, we also summarized NOX2 microglia activation that may be involved in the development and/or progressions of neurodegenerative diseases such as AD, PD, and ALS.

## 2. Neurotropism of SARS-CoV-2

Recent studies have confirmed the critical elements for cellular susceptibility to SARS-CoV-2 infections. Usually, SARS-CoV-2 can enter into host cells by binding to the Angiotensin-converting enzyme 2 (ACE2) receptor and transmembrane serine protease 2 (TMPRSS2) through the spike (S) glycoprotein located on the surface of the virus [13]. However, the distribution of ACE2 receptors in the host organism determines the viral tropism. ACE2 is a ubiquitous receptor widely expressed in alveolar epithelial cells of the lung, oral and nasopharyngeal mucosa, endothelium, and vascular smooth muscle cells in the brain, heart, kidneys, and bladder. The distribution of ACE2 in nervous tissue is poorly represented in neurons and microglia, instead, ACE2 mRNA has been found in the spinal cord. The function of ACE2 is to control inflammation and blood pressure [14,15,16,17,18]. As ACE2 is expressed in different organs, this could explain both the multiplicity and diversity of the symptoms associated with SARS-CoV-2 infection [19]. Although viral infection predominantly involves the respiratory system, the latest studies suggest that the virus can affect other organs such as the brain and nervous system. Indeed, the distribution of ACE2 receptors in the olfactory epithelium was observed, so SARS-CoV-2 could enter the brain through the olfactory nerve and olfactory bulb [20]. Furthermore, ACE2 has been detected in neurons and glial cells but also in brain structures including the brainstem, corpus striatum, cortex, and hypothalamus which could further explain the presence of the virus in the brain [21]. Noteworthy, the detection of SARS-CoV-2 in cerebrospinal fluid (CSF) samples from patients with COVID-19 proving its neurovirulence [22].

To explain the presence of the virus in the nervous system, some viral entry routes have been hypothesized. Especially, SARS-CoV-2 can spread in the CNS through two main routes, haematogenic and axonal transport. The hematogenous pathway allows SARS-CoV-2 to enter directly into the CNS through the endothelial cells of the blood–brain barrier (BBB), according to a mechanism whereby SARS-CoV-2 can bind ACE2 expressed in the capillary endothelium of BBB, or by infecting monocytes and macrophages to spread directly into the CNS through the BBB [23]. As regards axonal transport, it originates from the nasal cavity through the cribriform plate until it reaches the brainstem, involving different anatomical structures such as the nasal cavity, the olfactory nerve, the olfactory bulb, the piriform cortex, as well as the brainstem [24,25]. Moreover, the axonal transport could be implicated in respiratory insufficiency as the virus through the olfactory nerve and its subsequent progression in the rhino-encephalon could reach the respiratory centers of the brainstem modulating the control of the respiratory rate [26]. Furthermore, recent studies have shown that the entry of SARS-CoV-2 can be facilitated by the presence of the neuropilin-1 receptor (NRP1), expressed both in the human brain and in the olfactory epithelium [27].

## 3. Methodology

The purpose of this manuscript is to describe the SARS-CoV-2 neurotropism and its possible involvement in the activation of NOX2 in microglia. Furthermore, we provide an overview aimed to illustrate NOX2 activation as a possible cause of NDs such as PD, AD, and ALS. 

To select the studies, we performed a PubMed and Scopus search using the keywords. The following keyword combinations: SARS-CoV-2 and Microglia; Microglia and angiotensin 2 and NADPH oxidase; NOX2 and Parkinson’s disease; NOX2 and Alzheimer’s disease; NOX2 and Amyotrophic lateral sclerosis, were used to find suitable publications. We have considered articles published up to 14 April 2021. Ultimately, in this review, 53 studies were considered evaluating the relationship between SARS-CoV-2 infection and NOX2 activation in microglia, to evaluate its molecular mechanism underlying neuroinflammation to prevent the development of NDs.

## 4. Microglia Activation and Neuroinflammation

Recent investigations on COVID-19 patients have shown an increase in inflammatory mediators such as cytokines and chemokines believed to be responsible for the physiological alteration of the CNS [28]. These chemical mediators would be involved in both the activation of microglia and astrocytes, resulting in neuroinflammation [29]. Moreover, results from post-mortem neuropathological studies in COVID-19 patients showed that microglia activation and cytotoxic T lymphocyte infiltration were mostly represented in the brainstem, cerebellum areas [30], and also olfactory bulb and medulla oblongata [31]. 

The main role of activated microglia is to promote and exacerbate the inflammatory process through the production of cytokines such as TNFα, IL-1β, IL-6, compounds such as nitric oxide (NO), ROS, and reactive nitrogen species (RNS) which can cause neuronal damage and neurodegeneration [29,32].

The renin-angiotensin system (RAS) in the CNS is well represented and its components are present in glial cells such as astrocytes and microglia and in different areas of the brain such as the cortex and hippocampus [32]. Ang II is a key effector peptide of the RAS known to regulate blood pressure, behavior, central sympathetic activity. Nevertheless, Ang II can have deleterious effects as it contributes to the development of oxidative stress and inflammation [33].

SARS-CoV-2 infection can hinder the interaction with ACE2, leading to interaction with the Angiotensin receptor (AT1) [29]. Therefore, Ang II binds to AT1 and subsequently activates by promoting the change of microglia from immunoregulatory M2 to pro-inflammatory M1 phenotype [34]. Notably, M1 microglia phenotype is activated by compounds such as Toll-like receptor (TLR)-4 agonist, lipopolysaccharide (LPS), interferon (IFN)-γ, TNFα, and also by the increase of NOX levels [35].

NOX activation is not only concerned with free radical’s production such as superoxide anion but also involved in the regulation of Rho-kinase (ROCK) activity by inducing changes in the actin cytoskeleton and stimulating the migration of inflammatory cells. [36]. Additionally, ROCK activation induced by Ang II can lead to the TNF-α release from microglia, thereby stimulating neuronal NF-κB transcription and promoting both neurodegeneration and further microglial activation [37]. At the same time, ROCK is involved in NOX activation through p38 mitogen-activated protein kinase (MAPK). Although there is further evidence, some investigations prove that Ang II can activate both NOX and ROCK, thus suggesting both pathways have a common mechanism [36]. 

Moreover, it has been observed that Ang II-induced oxidative damage in microglia leads to degradation of microglia ferritin and consequent release of iron which can exacerbate oxidative damage [38]. Certainly, NOX2 activation is implicated in the pro-inflammatory response and represents also one of the major sources of ROS, the main inducers of aging and cell damage as well as NDs [39]. 

Furthermore, microglial activation can seriously compromise the functionality of the BBB facilitating viral entry [29] and contributing to the infiltration of peripheral immune cells [28]. Indeed, structural alteration of BBB following CNS infection in COVID-19 patients stimulated a potent neuroinflammatory response characterized by microglial activation [40]. Recently, Mishra et al. focused attention on the role of exosomes in microglia to identify pathways involved in the neuropathogenesis of SARS-CoV-2. In this regard, human microglia were treated with exosomes from HEK-293T cells transfected with S gene plasmids. The exosomal vesicles contained high levels of miR-148a and miR-590 that modulate gene expression of Ubiquitin Specific Peptidase 33 (*USP33*) and Interferon Regulatory Factor 9 (*IRF9*), which are found downregulated. MiR-590 can directly target IRF9 while miR-148a suppresses USP33 expression levels in human microglia. USP33 is a deubiquitinase that targets IRF9. Indeed, IRF9 downregulation causes harmful effects in microglia, promoting microglial activation and neuroinflammation. Hence, the disruption of the SP33-IRF9 axis promoted by SARS-CoV-2 infection would activate the inflammatory phenotype of microglia which over time can induce neurodegeneration [41]. This evidence was found in a patient with COVID-19 in whom a strong microglial activation was found within the white matter [42]. This information supports the hypothesis that SARS-CoV-2 infection may induce severe neurological repercussions related to its invasion in the nervous system [29]. 

## 5. NOX2 Increases Oxidative Stress

NOX enzymes are protein complexes that can cross the cell membrane transfer electrons to molecular oxygen to generate superoxide anion as well as ROS including hydrogen peroxide (H_2_O_2_) and hydroxyl radicals (OH^●^). The NOX family includes 7 isoforms such as NOX1-5 and Drosophilae harbor a dual oxidase (DUOX) 1 and 2, characterized by six transmembrane domains, one cytoplasmic NADPH binding site, and two heme-binding sites [43,44]. Furthermore, all NOX enzymes through both the flavine adenine dinucleotide (FAD) domain and two heme groups, can catalyze the transfer of two electrons from NADPH to molecular oxygen [45]. 

NOX2, the first NOX enzyme to be discovered, is widely expressed in phagocytes including neutrophils and microglia where it exerts a defensive action against pathogens, as well as in the proliferation and signaling cell [46,47]. 

NOX2, encoded in humans by the *NOX2* gene, is the catalytic subunit linked to the phagocyte membrane NADPH oxidase (PHOX). The NOX2 activation involves the binding with p22^PHOX^, a membrane protein that together with NOX2 was found in the phagosome [48,49]. Therefore, the binding of NOX2 with p22^PHOX^ leads to the formation of the heterodimer known as flavocytochrome b558 [50]. NOX2 activation requires the presence of cytosolic organizing and regulating proteins including p40^PHOX^, p47^PHOX^, p67^PHOX^, and the G-protein Rac [43]. Consequently, the NOX2 activation involves the translocation of p40^phox^, p47^phox^, p67^phox^, and Rac into a heterodimer cytochrome composed of gp91^PHOX^ and p22^PHOX^, as well as the subsequent conversion of molecular oxygen to superoxide anion. [51]. Probably, the NOX2 complex is activated through the phosphorylation of p47^PHOX^ in which several kinases have been involved as well as the replacement of guanine nucleotides on Rac [52]. P47^PHOX^ is found quiescent in the cytoplasm of microglia, as soon as activated it is phosphorylated and subjected to a conformational change that allows it to translocate on the membrane to bind membrane phospholipids including the phosphatidylinositol-3 kinase (PI3K) as well as the C-terminal of p22^PHOX^ characterized by proline residues [53,54]. Additionally, p47^PHOX^ can also activate the p67^PHOX^ subunit, which once active can bind Rac, probably through direct interaction with gp91^PHOX^ [55]. Therefore, both p47^PHOX^ and p67^PHOX^ are necessary to activate NOX2 at the microglial level [56]. In the same way, it was shown that the cytosolic subunit p40^PHOX^, although not directly required for enzymatic activity, is involved in the modulation of ROS generation [57,58]. 

NOX2 exerts its antimicrobial action by direct production of ROS, as well as through the activation of granular proteases and generation of neutrophil extracellular traps (NETs). [59]. In the same way, NOX2 can play an important role in the regulation of adaptive immunity, including both antigen presentation and lymphocyte response [59,60].

Under physiological conditions, NOX exerts several functions including host defense, signaling and differentiation cell as well as the regulation of gene expression. Additionally, it has been shown that increased NOX expression can contribute to the development and/or progression of neurodegenerative and cardiovascular diseases [43]. 

Notably, NOX2 can be involved in the pathogenesis of diseases in which inflammation plays an important role, including AD, PD, and ALS [60,61]. As, oxidative stress, inflammation, and microglial activation are common in the NDs, NOX2 could be a useful therapeutic target for their management. 

## 6. Role of NOX2 in Neurodegenerative Diseases

The NDs such as AD, PD, ALS represent a growing and frequent cause of disability in developed countries. The common cause of these pathologies is represented by oxidative stress which implementing with aging. The researcher has reached new goals as the role of NOX2 in neurodegeneration. Notably, NOX2 activation in the CNS may be involved in the development and progression of these disabilities. Furthermore, NOX2 activation can amplify the inflammatory process through both activation and differentiation of lymphocytes T [11]. NOX2 activation also contributes to promoting oxidative stress, since its activation induces superoxide production [62]. Given the above, NOX2 can be regarded as a novel therapeutic target to prevent and counteract neurodegeneration [63]. 

### 6.1. NOX2 in Parkinson’s Disease

The PD is a progressive ND characterized by progressive loss of dopaminergic neurons in the substantia nigra pars compacta (SNpc) of the brain, with a consequent reduction of dopamine (DA) levels. The hallmarks of PD are the aggregates of α-synuclein (α-syn) included in Lewy’s bodies in dopaminergic neurons in the SNpc and other areas of the brain [64]. The main disorders in PD are tremor, bradykinesia, rigidity, and balance disturbances and they influence the individuals’ quality of life [65]. Neuronal degeneration is promoted from several factors such as inflammation and oxidative stress. Most cases of PD (about 90%) are in the sporadic form. Instead, a small percentage (about 10%) represent a familial form, since genetic mutations such as Synuclein Alpha (*SNCA*), leucine-rich repeat kinase 2 (*LRRK2*), Parkin (*PRKN*), protein deglycase (*DJ-1*), phosphatase, and tensin homolog-induced putative kinase 1 (*PINK1*) are involved [66]. Recent studies have shown that PD patients may be more susceptible to SARS-CoV-2 infection as it compromises adaptive immunity. Furthermore, it has been hypothesized that this viral infection could induce the accumulation of α-synuclein, causing neurodegeneration. Furthermore, it has been hypothesized that this viral infection could induce the accumulation of α-synuclein, causing neurodegeneration. In this regard, it has been shown an increase of inflammation following the infiltration of SARS-CoV-2, which in turn can lead to the activation of endothelial cells in the pulmonary vascular system, as well as to the development of hypoxia. Consequently, respiratory hypoxia in COVID-19 patients could promote ROS formation and oxidative stress in the brain. Additionally, the increase of ROS levels could lead to an alteration of the redox balance in the mitochondria and thus promote apoptosis. Moreover, there is evidence that oxidative stress induced by increased ROS following hypoxia, may contribute to the death of dopaminergic neurons and thus promote PD [40].

The microglial activation and pro-inflammatory cytokines have been observed in the substantia nigra (SN) in patients with PD. Recent studies have shown that α-Syn is related to NOX2 activation, which in turn was able to induce the microglia activation, however, the mechanism is not yet known. Hou et al., investigated the role of integrins in NOX2 activation, especially in CD11b, the α chain of the αMβ2 integrin, expressed in immune cells as microglia. This experimental study was conducted on microglial cells treated with α-Syn (50, 100, and 200) nM useful for reproducing a PD model. Indeed, it was possible to observe the translocation of p47^PHOX^ (NOX2 subunit) in the membrane and consequently, an increase in ROS production which would represent a key step for NOX2 activation. Subsequently, α-Syn-induced microglia were treated with anti-CD11b antibodies showing neither p47^PHOX^ translocation nor ROS production. Likewise, as observed in vitro experiments, NOX2 did not activate in CD11b ^−^/^−^ compared to wild-type (WT) mice. Additionally, an increase in RhoA and ROCK levels was observed. To evaluate whether Rho-kinase was involved in NOX2 activation, α-Syn-induced microglia were transfected with RhoA siRNA. RhoA knockdown in microglia was able to reduce RhoA and ROCK expressions as well as inhibiting superoxide production and translocation of p47^PHOX^. Thus, in this study, it was demonstrated that CD11b might play a role in α-Syn-induced NOX2 activation [67]. In compliance with these results, another study has focused on the involvement of integrins in NOX2 activation and its possible implication in PD. To reproduce a PD model, primary midbrain neuron-glia cultures were exposed to active metabolite 2,5-hexanedione (HD) (8 mM). Zhang et al. observed a significant loss of dopaminergic neurons caused by the microglia activation. Indeed, Microglia-depleted cultures subjected to the same treatment did not show significant alteration in cell viability. Furthermore, the authors observed increased ROS levels in BV2 microglial cells exposed to 4, 8, and 16 mM (HD). To clarify whether superoxide was produced by NOX2, microglia cells were co-treated with apocynin suggesting that NOX2 was responsible for inducing ROS production and also the translocation of p47^PHOX^. Likewise, also in vivo was obtained an increase in p47^PHOX^ levels in the midbrain membranes of HD-treated mice compared to control. Instead, to assess whether αMβ2 integrin (also known as CD11b) was involved in NOX2 activation, HD-induced, microglia cells were co-treated with integrin inhibitors Arginylglycylaspartic acid (RGD) or anti-αM antibody. The findings showed that both the RGD and the anti-αM antibody were able to inhibit superoxide production and p47^PHOX^ translocation. Coherently, the same results were found in HD-treated mixed-glia cultures obtained from αMβ2-deficient (αM ^−^/^−^) mice Further demonstrating the implication of this receptor in NOX2 activation. Finally, the authors also assessed that the αMβ2-Src-ERK pathway would play a key role in NOX2 activation. Thus, HD-treated BV2 microglia were pre-treated with Src and ERK inhibitors, demonstrating that inhibition of the αMβ2-Src-ERK pathway had attenuated neurotoxicity in dopaminergic neurons [68]. Instead, Hou et al. also investigated NOX2 activation the potential role of complement receptor 3 (CR3) in NOX2 activation of microglia cells after exposure to pesticides. BV2 microglial cells were treated with a combination of paraquat and maneb (P+M), respectively 10 + 0.6 μM concentrations for inducing dopaminergic neurodegeneration. In this way, increased ROS levels and p47^PHOX^ translocation were observed, demonstrating the NOX2 activation. Conversely, pretreatment with NOX2 inhibitors such as apocynin and diphenyleneiodonium (DPI) in BV2 microglia demonstrating that ROS levels and p47^PHOX^ translocation were induced by NOX2. Subsequently, the CR3 receptor (integrins) role was studied, thus its inhibition obtained with RGD-pretreatment in BV2 microglia cells. The results decreased both superoxide production and translocation of p47^PHOX^, supporting the hypothesis of integrins involvement in NOX2 activation. Consequently, integrin inactivation impacted the Src-ERK pathway. Indeed, both inhibitions of Src and ERK reduced the translocation of p47^PHOX^. Instead, immunofluorescence analysis was performed to detect the microglia activation and its morphology using an anti-Iba-1 antibody, a microglia marker. The findings revealed greater cellular bodies, reduction in the number of dopaminergic neurons, and major reactivity to Iba-1 antibody after P+M-treatment in WT than CR3 ^−^/^−^ mice. Based on these results, it was possible to confirm that the C3-Src-ERK pathway could be strongly implicated in NOX2 activation [69]. In compliance with the previous study, P+M treatment (10 + 30 mg/kg, respectively) was also administered to male C57BL/6J mice for inducing dopaminergic neurotoxicity after 2, 4, and 6 weeks of exposure. The dopaminergic neurodegeneration was increased in a time-dependent manner. Moreover, P+M treatment was able to increase α-synuclein oligomer levels. Therefore, the authors administered taurine (150 mg/kg) to mice treated with P+M for 6 weeks, obtaining encouraging results on the improvement of both dopaminergic neuron viability and α-synuclein expression. Furthermore, taurine has contributed to attenuate microglial activation and p47^PHOX^ and gp91^PHOX^ levels after P+M exposure in the midbrain of mice. NOX2 could play a role not only in inflammation but would be able to induce microglia polarization through the activation of the NF-Κb pathway. However, taurine was able to mitigate dopaminergic neurodegeneration through the reduction of α-synuclein levels and NOX2 activation after exposure to P + M [70]. As widely known, the α-Syn aggregation was implicated in ND, including PD. Several studies have shown that α-synuclein mutations such as A30P and A53T resulted in increased activation of microglia, resulting in severe loss of dopaminergic neurons. For this reason, Wang et al. experimented with a neuron-glia culture exposed to A29-V40 peptide (α-synuclein mutations) or phorbol 12-myristate 13-acetate (PMA) to reproduce PD. These cultures were co-treated with apocynin to inhibit NOX2, and the co-treatment has contributed to restoring the viability of dopaminergic neurons and their ability DA uptake. Nevertheless, NOX2 inhibition is less efficient in neuronal cells LPS-treated, indicating that other factors could promote neurotoxicity besides NOX2. Further comparison was made between neurons-astrocytes and neuron-glia cultures. Initially, neuron-astrocytes culture treated with A29-V40 did not show any neuronal damage. However, the addition of microglia to the latter culture showed a significant loss of DA uptake. Instead, gp91^PHOX −^/^−^ microglia were added to the neuron-astrocytes culture treated with A29-V40, demonstrating that this peptide did not compromise dopaminergic neurons. Based on the results obtained, NOX2 activity in microglia promoted A29-V40-induced neurotoxicity. Furthermore, both PMA and A29-V40 treatment had induced translocation of p47^PHOX^ and p67^PHOX^ in rat microglia-derived highly aggressively proliferating immortalized (HAPI) cells. A29-V40 peptide could bind gp91^PHOX^ on the surface and activate increasing superoxide levels. Hence, the superoxide could be converted in H_2_O_2_ by superoxide dismutase (SOD), then H_2_O_2_ could cross the membrane and subsequently promote phosphorylation. To assess whether treatment with PMA or A29-V40 could affect p47 ^PHOX^ and p67^PHOX^ phosphorylation, the microglia culture was pretreated with SOD and catalase or H_2_O_2_. In consideration of the results, H_2_O_2_-induced gp91^PHOX −^/^−^ microglia promoted the phosphorylation of p47^PHOX^ and Erk1/2 demonstrating that several factors could affect NOX2 activation. Likewise, microglia activation after A29-V40 peptide injection was evaluated in vivo. As evidence, activated microglia and increased malondialdehyde (MDA) levels were observed in WT mice rather than gp91^PHOX −^/^−^ mice. These results suggested that α-Syn could both activate NOX2 and promote microglial activation leading to neuronal damage [71]. The effects of simultaneous administration of LPS and α-Syn were evaluated in both in vivo and in vitro, to investigate the neurodegenerative mechanisms in the PD. Zhang et al. observed severe loss of dopaminergic neurons and decreased DA uptake in rat primary midbrain neuron-glia cultures treated with LPS (0.5 ng/mL) + α-Syn (20 nmol/L). It was interesting to observe that the co-treatment induced a less DA uptake in neuron-microglia culture compared to neuron-astroglia culture, thus demonstrating the role of microglia in promoting neurotoxicity. In addition, an increase in ROS levels was observed, therefore the role of NOX2 as a cause of increased ROS levels was investigated. The in vivo experiment was conducted in which NOX2^+^/^+^ and NOX2^−^/^−^ mice were compared to highlight different factors after administration of the LPS (0.2 ug/uL) + syn (0.0125 ug/uL) treatment in SN. Interestingly, a decrease in both dopaminergic levels and DA uptake was found in NOX2^+^/^+^ mice, conversely, both the number of microglia and the ROS levels increased. The same results were found in rat primary midbrain neuron-glia cultures obtained from NOX2^+^/^+^ and NOX2^−^/^−^ mice. However, p47^PHOX^ and gp91^PHOX^ were increased in LPS + syn treatment, demonstrating their involvement in NOX2 activation. Instead, DPI pre-treatment (NOX2 inhibitor) improved the viability of dopaminergic neurons, moreover, microglia and ROS were reduced. Furthermore, the combined treatment in microglia increased both mRNA expression and levels of Protein Kinase C (PKC-δ), p38, ERK, and NF-ΚB, thus ex-plaining that NOX2 promoted neuroinflammation [72]. Additionally, Jiang et al., evaluated the pharmacological proprieties of clozapine to reduce the inflammatory state induced by NOX2 activation. The neuroprotective effects of clozapine were investigated in neuron-glia mixed culture cells after induced LPS or 1-methyl-4-phenylpyridinium (MPP^+^) to explore their role in neurotoxic conditions. To evaluate the efficacy of pre-treatment with clozapine N-oxide (CNO) or N-Desmethylclozapine (NDC) for 30 min before LPS (15 ng/mL) or MPP^+^ (0.25 μM) treatment. CNO (0.01 μM) or NDC (0.1 μM) pretreatment demonstrated its neuroprotective effects as an increase in both DA uptake and viability of dopaminergic neurons. These compounds also reduced Iba-1 and CD11b levels in LPS-induced microglia. Of note, LPS treatment induced the release of inflammatory compounds such as superoxide, TNF-α, and NO. However, superoxide release was mitigated in pre-treatment with CNO or NDC, modulating neuroinflammation. To investigate the role of NOX2, dopaminergic neurons obtained in gp91^PHOX^-deficient mice were compared with WT (gp91^PHOX +^/^+^) mice and treated with LPS. Dopaminergic neurons of gp91^PHOX^-deficient mice after LPS-treatment showed no difference in DA uptake. Further demonstration of NOX2 involvement after the LPS-treatment was given by the inhibition of p47^PHOX^ translocation caused by CNO and NDC in microglia HAPI cells. To prove the neuroprotective effects of these compounds, male C57BL/6J mice were treated with MPP^+^ (20 mg/kg) for 6 days and pretreated with CNO or clozapine (1 mg/kg) for 21 days. In line with previous findings, CNO reduced neuronal damage and ameliorated motor deficit MPP+-induced. Therefore, these pharmaceutical compounds have demonstrated their beneficial properties for protection against neurotoxicity useful for the management of NDs such as PD [73]. To illustrate the implications of both microglia and NOX activation in NDs such as PD, a group of researchers conducted an in vivo experiment. In this study, a PD model was induced by intraperitoneal (i.p) injection of LPS (5 mg/kg) in male C57BL/6J (NOX2^+^/^+^) and Cybb (NOX2^−^/^−^) mice which served to promote neurodegeneration and increase oxidative stress. Immunohistochemical analysis was performed through which the loss of dopaminergic neurons was observed in NOX2^+^/^+^ mice 10 months after treatment with LPS in SNpc. In addition, it was observed an upregulated expression of Iba-1 mRNA, a characteristic morphology of activated microglia, and a progressive increase in the number of activated microglia in LPS-treated NOX2^+^/^+^ mice after 10 months. It was seen that the mice of the control group treated with saline only showed an increase in activated microglia 2, 7, and 10 months later, demonstrating that microglia activation could depend on both aging and exposure to toxic compounds. Besides, LPS-treatment in C57BL/6J (NOX2^+^/^+^) mice promoted both mRNA NOX2 expression and release of ROS in a time-dependent manner. Consequently, the authors have investigated the cause of NOX2 induction. For this reason, they used anti-gp91^PHOX^, anti-Iba, anti-TH (a marker of the dopaminergic neuron), and anti-GFAP (a marker of astrocyte) antibodies in the brain tissue of hydroethidine-treated mice (10 mg/kg, i.p.) 10 months after a single dose of LPS-injection. The results highlighted that NOX2 and ROS were prevalently co-localized in both microglia and dopaminergic neurons. To confirm the implication of NOX2 in neurodegeneration, DPI (3 mg/kg, ip), was administered to mice that have reduced both ROS and inflammatory cytokines such as TNFα, the monocyte protein chemoattractant-1 (MCP-1), and LPS-induced IL-1β. Hence, NOX2 activation was responsible for microglia activation and, subsequently, for the release of inflammatory factors, thus promoting neurodegeneration [74]. Gao et al. performed an investigation based on the anti-inflammatory proprieties of IL-10 to discover the mechanism for preventing neuroinflammation and neurodegeneration. The investigation focused on the activation mechanism of NLR family pyrin domain containing 3 (NLRP3) and its influence on both pro-caspase-1 and pro-IL-1β cleavage. As observed, pretreatment of IL-10 in both WT and IL10^−^/^−^ microglia culture reduced NLRP3, IL-1β, and caspase-1 mRNA expression. The same results were obtained also in vivo, demonstrating the ability of IL-10 to modulate NLRP3 activation. Recent investigations have shown that ROS were able to induce NLRP3 inflammasome activation. To demonstrate the involvement of ROS in this process, both WT and IL10^−^/^−^ microglia culture LPS-treated were compared. It was examined whether NOX2 could be a possible source of ROS. The translocation of p47^PHOX^ which is reflected in NOX2 activation was observed in the culture of IL10^−^/^−^ microglia treated with LPS. The neuro-degenerative effects of LPS treatment were attenuated through the administration of NOX2 inhibitors such as DPI or apocynin, which had reduced the cleavage of pro-caspase-1 and pro-IL-1β in LPS-induced IL10^−^/^−^ mice. Therefore, it was possible to deduce that NOX2 activation and the consequent ROS increase were responsible for the activation of the NLPR3 inflammasome. The current study allowed us to highlight that IL-10 was able to suppress LPS-induced ROS-NOX2 and to block the activation of NLPR3, preventing neuroinflammation. Given the above, it was possible to demonstrate that IL-10 exerts neuroprotective and anti-inflammatory actions [75]. Recently, the possible connection between NOX2 and Nucleotide-binding oligomerization domain-containing protein (NOD2) has been investigated aimed to observe their role in the pathogenesis of PD. In this context, male C57BL/6J and NOD2 knockout (NOD2^−^/^−^) mice were treated with 6-hydroxydopamine (6-OHDA) (2 μL) to reproduce the PD model. It was observed a progressive reduction of tyrosine hydroxylase (TH) was observed in both striatum and SN which is reflected in a loss of DA after treatment. Furthermore, the results also showed NOD2 upregulation in WT mice, especially in microglia. Cheng et al. investigated that NOD2 was related to the apoptotic process. Indeed, an increase in both the number of apoptotic cells and apoptotic proteins such as BAX, Bcl-2, caspase-3, and cytochrome C were observed after 6-OHDA or muramyl dipeptide (MDP) treatment in WT. In line with these results, it was observed that activation of both microglia and astroglia were more represented in WT compared to NOD2^−^/^−^ mice, thus explaining neuroinflammation. Likewise, it emerged from the increase in monocyte chemoattractant protein-1 (MCP-1), IL-6, TNF-α, and IL-12p70 in WT mice. In this context, gp91^PHOX^ expression was observed in NOX2 and ROS production in SN after 6-OHDA injection in a time-dependent manner. In vitro, the results showed an increased NOX2 and ROS levels in BV2 microglial cells in a time-dependent manner. In the same way, an increase of inflammatory compounds such as NOX2, NOD2, inducible nitric oxidase synthase (iNOS) was observed in 6-OHDA induced microglia. To explain the role of NOX2 in NOD2 mediated-neurotoxicity, a viability assay was performed, in which SH-SY5Y and BV2 microglia cells were co-incubated and subsequently treated with 6-OHDA or MDP. This treatment demonstrated that microglial activation induced neurotoxicity. Furthermore, the neuroprotective effects of apocynin have been demonstrated since the latter increased the viability of dopaminergic neurons after 6-OHDA or MDP treatment. Interestingly, this study suggested that both NOX2 and NOD2 promoted neurodegeneration, especially in the PD model [76]. In compliance with the previous study, also Hernandes et al. wanted to examine the effects of 6-OHDA treatment in mice (C57BL/6) and gp91^PHOX −^/^−^ useful for reproducing the PD model. The first evaluation was made on NOX mRNA expression after the 6-OHDA treatment, in which an upregulated NOX2 expression was found both in SN and striatum. Although no translocation of NOX2 subunits was observed, it was detected Ras-related C3 botulinum toxin substrate 1(Rac1), an important protein involved in NOX activation. Instead, it was interesting to observe that 6-OHDA treatment in the striatum of gp91^PHOX−^/^−^ mice significantly reduced the number of dopaminergic neurons, emphasizing NOX2-mediated neurotoxicity. To assess microglia-promoted neurotoxicity, 6-OHDA-induced mice were co-treated with minocycline, a microglial inhibitor with both anti-inflammatory and antimicrobial proprieties. Treatment with minocycline increased the viability of dopaminergic neurons in WT while it was able to promote neurodegeneration in gp91^PHOX +^/^+^ mice. On other hand, more pro-inflammatory cytokines and chemokines were found in WT mice treated with 6-OHDA but were not increased after co-treatment with minocycline. Furthermore, neurotoxic treatment promoted a significant inflammatory response through a major re-lease of TNF-α in WT compared to gp91^PHOX−^/^−^ mice, further supporting NOX2 involvement [77].

Experimental studies have shown that iron deposits have been observed in the SN of patients with PD. Indeed, Fe^2+^ would have the ability to induce neurotoxicity, so Zhang et al. wanted to investigate the implications of that in the pathogenesis of PD. The authors examined the mechanism of microglia activation in rat primary midbrain neuron–microglia–astroglia cultures after Fe^2+^-treatment (5, 25, and 100) μM. In this experiment, exposure to Fe^2+^ was responsible for the loss of dopaminergic neurons and has induced morphological changes in the midbrain neurons as they exhibited smaller cellular bodies. Likewise, Fe^2+^-treatment in rat primary microglia cultures produced an increase in superoxide anion supported by morphologic changes in microglia. Therefore, it was interesting to verify that the same results were observed in primary microglia culture obtained from NOX2^+^/^+^ and NOX2^−^/^−^ mice treated with Fe^2+^. Indeed, an increase in both superoxide levels and the number of activated microglia in NOX2^+^/^+^ was shown after Fe^2+^ exposure. Nevertheless, Fe^2+^-treated NOX2^−^/^−^ mice showed less dopaminergic neuron loss demonstrating their resistance to treatment. Consequently, to confirm the activation of NOX2, this culture was pre-treated with DPI showing a reduction in both the activated microglia and the loss of dopaminergic neurons. Furthermore, the evidence showed that Fe^2+^ had significantly increased both p47^PHOX^ and gp91^PHOX^ expression (NOX2 subunits) and their increased expressions suggested that Fe^2+^-induced NOX2 activation. Finally, it was observed an increase in mRNA expression and protein levels of p38, ERK 1/2, and JNK supporting that Fe^2+^ exposure could promote neuroinflammation. Therefore, iron has been shown to induce neurotoxicity by causing both the loss of dopaminergic neurons and the activation of microglia [78].

The substance P (SP) is widely studied for its implication in the pathophysiology of PD. Wang et al., examined the role of SP in both in vivo and in vitro, demonstrating its correlation in neurodegeneration. For this reason, male WT (C57BL/6J), SP-deficient (TAC1^−^/^−^), and SP receptor-deficient (NK1R^−^/^−^) mice were treated with LPS or MPTP to induce neurotoxicity. Immunostaining was performed 10 months after treatment with LPS or MPTP in WT and TAC1^−^/^−^ mice. This treatment highlighted a progressive loss of dopaminergic neurons in WT compared to TAC1^−^/^−^ mice. Furthermore, an increase of TNF-α, iNOS, and MCP-1 expressions were observed in WT mice. To uncover microglia activation in SN after LPS-treatment, Iba-1 and CD11b were found downregulated in the transgenic mouse, suggesting that SP stimulated the neuroinflammation process in WT mice. Instead, the role of NOX2 in enhancing the effects of combined treatment of subpicomolar SP with LPS or MPTP in WT or NOX2-deficient (gp91^PHOX −^/^−^) glia-neuron cultures obtained from mice was demonstrated. In WT culture treated with SP + LPS or SP + MPP^+^ was observed a significant loss of dopaminergic neurons compared to gp91^PHOX −^/^−^ culture; thus it was inferred that NOX2 may play a role in promoting neurotoxicity. Moreover, the treatment with LPS + SP was able to promote p47^PHOX^ and p67^PHOX^ translocation. In addition, it was also observed that gp91^PHOX^ was identified as a target of SP further demonstrating NOX2 activation. Obviously, NOX2 activation had generated ROS, which in turn stimulated the activation of MAPK and NF-Κb pathways. As evidence, subpicomolar SP was found to have the ability to promote MAPK and NF-Κb activation in microglia in a NOX2-dependent manner. Pretreatment with DPI reduced ROS production and, subsequently demonstrated that MAPK and NF-Κb pathways were activated following NOX2 activation in microglia after treatment. Therefore, it was possible to define that both SP and NOX2 modulate neuroinflammation, thus playing a role in the viability of dopaminergic neurons [79]. Recently, preclinical observations have focused on the study of glucose metabolism in PD patients. Metabolic dysfunction is caused by alterations in the pentose phosphate pathway, the main pathway used by neuronal cells to metabolize glucose. Several studies have shown an increase in glucose-6-phosphatase-dehydrogenase (G6PD) levels in PD models. For this reason, Tu et al. conducted both in vivo and in vitro experiments to investigate the involvement of G6PD in neurodegeneration. It was performed in an in vivo experiment using both C57BL/6 J mice and A53T mutant α-synuclein transgenic mice. C57BL/6 J mice were administered with a single i.p. or intranigral injection of LPS (5 mg/kg) or subcutaneous injection of MPTP (15 mg/kg) for 6 days. The treatment has shown increased levels in both gp91^PHOX^ and G6PD were observed in LPS-induced mice resulting in a reduction in both TH and Iba-1 levels. Moreover, upregulation of G6PD was observed mainly in microglia after treatment with LPS or MPTP. Instead, it was found in the midbrain of A53T mutant α-synuclein transgenic mice increased in both gp91^PHOX^ and G6PD levels compared to WT mice. Subsequently, the enzymatic activity of G6PD and the levels of NADPH in neuron-glia culture treated with saline or LPS were evaluated to demonstrate a possible implication of NOX2 activation. Indeed, neuron-glia culture treated with LPS demonstrated an increase of NADPH levels and G6PD activity after LPS-treatment, so it could be the promoter of NOX2 activation that induces neuroinflammation. To observe that G6PD could affect NOX2 activation, G6PD was inhibited. Indeed, pre-treatment with the G6PD inhibitor reduced ROS increases as well as oxidative stress. Likewise, a reduction in activation of the NF-Kb pathway demonstrating that G6PD inhibition could prevent neuroinflammation. Substantially, the increase of both G6PD and NOX2 in microglia implemented the oxidative stress, the NF-Kb translocation, and subsequent neurodegeneration. Therefore, the inhibition of G6PD limited both oxidative stress and inflammation in the microglia [63].

Exposure to environmental factors such as 6-OHDA, HD, MPTP, or genetic factors such as α-syn, A29-V40 induced the loss of dopaminergic neurons and the reduction of DA uptake, hallmarks of PD. In these experimental models, the studies focused mainly on the activation of microglia which activated NOX2 by promoting neuroinflammation through the release of ROS and pro-inflammatory cytokines (Table 1). To demonstrate the involvement of NOX2, pretreatments with apocynin, DPI were carried out demonstrating that their inhibitory action towards NOX2 would attenuate the release of ROS and reduce the translocation of NOX2 subunits such as p47^PHOX^ and p67^PHOX^.

Furthermore, clozapine, IL-10, taurine reduced the inflammatory state promoted following exposure to neurotoxic compounds and by-products of oxidizing and inflammatory compounds released by NOX2 activation. Although further investigations are needed, these studies demonstrate that NOX2 in microglia is involved in the signaling pathways underlying the inflammatory response. NOX2 would be involved in the toxicity of dopaminergic neurons therefore its inhibition in microglia could provide neuroprotection in the context of PD.

### 6.2. NOX2 in Alzheimer’s Disease

AD is a neurological disease characterized by cognitive impairment, progressive loss of memory, and brain function that can lead to death [80]. The hallmarks of AD are the extracellular aggregates of beta-amyloid (Aβ) peptides leading to the formation of amyloid plaques and intracellular accumulation of hyperphosphorylated tau protein, which in turn leads to the formation of neurofibrillary tangles (NFTs). Aβ peptides originate by the enzyme proteolysis of the amyloid precursor protein (APP), this latter is cleaved by β and γ-secretase to generate a peptide of 40 amino acids and a peptide of 42, known as, Aβ_1–40_ and Aβ_1–42_ [81,82].

NDs such as AD are characterized by neuroinflammation and oxidative stress which can promote disease progression. Consequently, the protein aggregation and neuronal damage can lead to microglial activation that induces ROS formation and neuroinflammation [83]. 

It has been demonstrated that TGF-β in the hippocampus regulates the microglial activity, it also plays an important role in neuroinflammation by controlling the change of microglia from a protective to harmful state [84,85,86]. Moreover, with aging, it has been shown a further increase of TGF-β levels as well as the activation of pathways related to inflammation, including ERK)/MAPK, p38 MAPK, c-Jun N-terminal kinase (JNK), and PI3K [87], that a decay of mitochondrial and lysosomal activity, as well as an increase of ROS that induces an exacerbation of neuroinflammation [88,89]. Consequently, changes in the CNS related to aging can lead to inflammation, an increase of BBB permeability, and ROS production thus neuronal degeneration such as AD [90]. Noteworthy as microglia have been found in AD patients, both in amyloid plaques and neurofibrillary tangles, where once activated they produce neurotoxic factors including superoxide, nitric oxide, and TNFα [91,92]. The important role of Aβ in plaque formation was explained by the amyloid hypothesis, which reported that microglia-mediated neurotoxicity is a key mechanism in disease progression [92,93]. Based on this, it was demonstrated that Aβ recruits and activates microglia [94]. Indeed, microglia can be activated by fibrillar Aβ through the receptor complex formed both by TLR2, TLR4, and also CD14 co-receptor, to activate intracellular signaling as well as ROS production and phagocytosis [95], by increasing oxidative stress in the brain of AD patients [96]. Furthermore, it has been hypothesized that SARS-CoV-2 infection may stimulate interferons, which also play a role in the development of AD. In fact, the latter would stimulate the microglia by activating a storm of cytokines that would lead to the degeneration of the synapses. [40]. We hypothesize persistent systemic inflammation caused by SARS-CoV-2 may trigger microglial activation, mediated by the massive release of pro-inflammatory cytokines secreted in response to this viral infection, aggravating the neurodegeneration leading to AD.

Geng et al. demonstrated both in vitro and in vivo the role of NOX2 in ROS production, as well as the signaling involved in the regulation of the microglial activation. BV2 microglial cells treated with Aβ_42_ (0.1–10 µM) for 24 h showed a significant increase of NOX2 and ROS. Moreover, activation of oxidative stress and related pathways including ERK1/2 and p38MAPK was evaluated by Western Blot and immunofluorescence analysis. Consequently, a significant increase in IL-1β and neuroinflammation has also been reported. While, treatment with NOX2 inhibitors including apocynin (20 μM) or NOX2tat (10 μM) significantly reduced ROS production, as well as confirming the important role of NOX2 in oxidative stress. 

Additionally, ROS production was completely abolished using Tiron (a ROS scavenger) and also confirming that TNF-α induces microglial proliferation through its action on microglial NOX. To evaluate both the role of NOX2 and ROS in Aβ-induced microgliosis and the effects of inflammation in aging, the same authors performed the study on mesencephalic sections of both WT C57BL/6 and NOX2-Knockout (KO) mice, respectively of young age (3–4 months) and elderly (20–22 months). In vivo results showed a greater Aβ aggregation, as well as high levels of ROS produced by NOX2, and also an increase in microgliosis and IL-1β in elderly WT mice (20–22 months) compared to young WT mice (3–4 months). In the same way, the results of the study were also confirmed in post-mortem mesencephalic samples of young (25–38 years) and elderly (61–85 years) humans without NDs. Indeed, it has been shown that in elderly human brains, in addition to the increase in ROS, also an increase in microglia and expression of microglial NOX2, as well as an increase of IL-1β and activation of ERK1/2 signaling. Therefore, the study performed in all the different models has evaluated and confirmed the important role played by NOX2 in Aβ-mediated microglial activation, as well as the effects of ROS produced by NOX2 activation in aging. Similarly, inhibition or KO of NOX2, as well as the inhibition of ROS production NOX2-induced, has been shown to protect the brain from aging related to oxidative damage, inflammation, and microglial alterations [7].

Therefore, studying and realizing compounds capable of both avoiding the assembly of NOX2 and stabilizing free radicals, could be a useful strategy to counteract the oxidative stress as well as the neurodegeneration that characterizes the AD. Indeed, it has been shown that apocynin, a NOX2 inhibitor it was capable to prevent the translocation of the p47^PHOX^ and p67^PHOX^ subunits, as well as reducing the damage produced by oxidative stress. In addition, it was reported that apocynin can also inhibit enzymes such as ROCK, which is involved in the adhesion, morphology, and motility of cells [97].

ROCK is associated with NOX; indeed, it has been shown that the phosphorylation of ROCK-I through Rac, induces the assembly of NOX2. As they are involved in the growth and retraction of neurites, both the ROCK-I and NOX2 pathways are interesting for investigating neurological diseases including AD. Indeed, it has been shown that the overexpression of NOX2 and ROCK-I in microglia can contribute to the progression of neuronal damage, as well as to the development of the disease. Therefore, both inhibition of ROCK-I and NOX2 pathways may be a useful strategy for counteracting the neuroinflammation in neurological diseases [98].

Alokam et al. evaluated the biological activity of 18 new molecules potentially capable of inhibiting ROCK-I and NOX2. The authors with computational methods combined between molecular docking and pharmacophore have realized ROCK-I and NOX2 inhibitors through information obtained by ligand-bound protein. The pharmacophore model for ROCK-I was performed using fasudil at a dose approximately of (1µM) which, thanks to its crystalline structure, proves to be a potent inhibitor of ROCK in human neuroblastoma cells. The results of the study demonstrated that in human neuroblastoma cells the compound 3 was the most active compared to fasudil, indeed it has reduced the inflammation measured by the decrease of pro-inflammatory mediators including IL-6, IL-1β, and TNFα. Therefore, the study showed that the realizing of ROCK-I and NOX2 inhibitory compounds could be useful for the treatment of neurological diseases such as AD [99].

The importance of NOX2, as well as the confirmation of its role in oxidative stress, has also been demonstrated by Wilkinson et al., in B6-R1.40 transgenic mice and human THP-1 monocytes. The authors showed that chronic treatment for 9 months of ∼5 g/day/animal of rodent food enriched with ibuprofen in a final dosage of 62.5 mg/kg/day has reduced Aβ aggregation in the brain in mice, evaluated by thioflavin S positivity. Moreover, the chronic administration of ibuprofen has led to reducing both microglial activation and oxidative stress measured by the decrease of 4-hydroxynonenal levels. Although there are several sources of microglial ROS including NOX, cyclooxygenase (COX), xanthine oxidase, and lipoxygenase, the primary source of ROS has been shown to be attributable to Aβ-induced NOX2 activation in the brains of AD patients. To evaluate the effects of ibuprofen on NOX2, the authors demonstrated that the pretreatment with racemic ibuprofen for 1 h before fibrillar treatment Aβ_22–35_ (60 μM) in primary murine microglia reduced the intracellular production of superoxide Aβ-induced. Furthermore, Wilkinson et al., also performed the study on human THP-1 monocytes where they observed the same response obtained in primary microglia. As both function and assembly of NOX2 depend on Vav phosphorylation induced by Aβ aggregates, ibuprofen could inhibit Vav phosphorylation and hence NOX2, as demonstrated by ibuprofen pretreatment in human THP-1 monocytes. Therefore, these results suggest that ibuprofen could inhibit NOX activation and ROS production, thus leading to the reduction of damage oxidative stress-induced [100].

The cytotoxicity of Aβ aggregates, as well as the increase in the ROS production and the mechanism related to NOX2 activation, was evaluated by Carrano et al., in endothelial cells of the brain. The authors performed the study on brain samples from patients with capillary cerebral amyloid angiopathy, a disease that occurs in most AD patients characterized by Aβ aggregates in the cerebral arteries and capillaries. As for how the cerebral amyloid angiopathy is related to AD not yet fully known, the authors hypothesized that Aβ aggregation in capillaries may involve the integrity and functionality of BBB, dysfunction of which may represent a pathological event associated with NDs. Indeed, it has been suggested that ROS, especially those produced by NOX2 in response to Aβ aggregation, can modulate the integrity of BBB through the interruption of tight junctions. In this regard, the presence of Aβ aggregation in the capillaries was related both to an increase in the microglial NOX-2 activity and ROS production which in turn led to the loss of occludins and claudins, as well as to the loss of cellular adhesion. Carrano et al., performed an in vitro study using a human cerebral microvascular endothelial cell line (hCMEC/D3) which was administered synthetic Aβ_1–42_ at different concentrations. Indeed, it was shown that the treatment with Aβ (10 μM) for 24 h induced cell death. While the treatment with Aβ_1–42_ (1 μM) for 24 h led to a reduction in mitochondrial activity as well as an increase in H_2_O_2_ in a dose-dependent manner. Consequently, the presence of H_2_O_2_ led the authors to investigate the involvement of NOX2 and xanthine oxidase in the ROS production Aβ-mediated. Indeed, before treatment with Aβ_1–42_, the hCMEC/D3 cells were pretreated at concentrations (10 nM and 100 nM) for 24 h with different NOX2 inhibitors including DPI and allopurinol. The results of the study showed that all antioxidants were able to preserve hCMEC/D3 cells from cytotoxicity Aβ_1–42_-induced. Moreover, the allopurinol pretreatment 2 h before of inducing model restored both occludin and claudin expression levels, as well as confirming that ROS production Aβ-mediated is also involved in the alterations of tight junctions. Further confirmations of the cytotoxic effects of Aβ in capillaries were shown by upregulation of Receptor for advanced glycation end products (RAGE), which can transport Aβ across BBB. Additionally, to carrying Aβ into the brain, RAGE through NOX-2 activation can induce ROS production. Moreover, it was shown that RAGE blockade by anti-RAGE blocking antibody has prevented the reduction of cell viability Aβ-induced. Therefore, the study results demonstrated that NOX2 inhibitors, exogenous antioxidants, and RAGE inhibitors could be useful to reduce the cytotoxicity Aβ-induced and neuronal damage related to its [101]. 

Moreover, increased levels of C-X-C motif chemokine ligand 1 (CXCL1), the latter widely expressed in the CNS and activated when it binds to the CXCR2 receptor have also been demonstrated in AD patients. CXCL1 once bound to its receptor can activate PI3K/Akt and ERK/MAPK signaling, thus contributing to inflammatory responses and cell cycle. Shang et al., both in vivo and in vitro investigated the effects of CXCL1 on neural stem cells in the subventricular area. The authors showed elevated CXCL1 levels in CSF APP/PS1 mice of 4,8,12 months compared to the WT control mice of the same-aged. Likewise, a greater increase of CXCL1 levels was reported in 12-month-old APP/PS1 mice compared to 4-month-old APP/PS1 mice and also compared to wild-type mice. Therefore, the results obtained suggested that CXCL1 levels in CSF could be disease markers in these animals. CXCL1 is expressed in microglia, macrophages, oligodendrocytes, and neurons. To simulate and investigate the activation of macrophages and microglia during the neuroinflammation, the authors used respectively RAW264.7 and BV2 cells treated with LPS or Adenosine triphosphate (ATP) (1 mM or 5 mM) alone or combined. Indeed, it has been shown that in RAW264.7 cells the LPS + ATP treatment led to a significant increase in CXCL1 compared to RAW264.7 cells treated with LPS alone. Moreover, it was also observed that treatment with LPS and ATP (1 mM or 5 mM) in BV2 microglial cells had no significant effect on CXCL1 synthesis and secretion. Therefore, these results suggested that macrophages may be a major source of CXCL1 in APP/PS1 mice. Additionally, CXCL1 has also been reported to promote both proliferation and differentiation of neural stem cells. Indeed, it has been shown that infusion of CXCL1 (200 ng per mouse) into the lateral ventricle led to a significant increase in the cell proliferation assessed by nestin positivity. The same results were also confirmed in vitro, in neural stem cells treated with CXCL1 (100 ng/mL) for 24 h. Moreover, CXCL1 treatment has also been shown to inhibit the differentiation of neural stem cells into astrocytes, but not their differentiation into neurons. To understand how CXCL1 exerts these effects, Shang et al., demonstrated that CXCL1 promotes the proliferation of neuronal stem cells through the production of ROS as well as by the activation of PI3K/Akt signaling. Furthermore, it was shown that treatment with CXCL1 (100 ng/mL) for 24 h led to an increase in both ROS and NOX2. Confirmation of NOX2 involvement was performed in vivo, indeed treatment with apocynin (30 nmol per mouse) before the CXCL1 (200 ng per mouse) administration has inhibited CXCL1 in promoting the proliferation of neural stem cells. In conclusion, the study showed that in APP/PS1 mice CXCL1 originating from macrophages can favor the proliferation of neural stem cells in the subventricular area through the generation of ROS [102].

In vitro and in vivo studies have shown that Aβ peptides can promote NOX2 activation, therefore a direct correlation with the deposition of Aβ, dependent on both aging and NOX2 activity (Table 2). Preclinical studies demonstrated that exposure of microglial cultures with Aβ peptides resulted in NOX2 activation with a consequent increase in ROS, ERK 1/2, and p38 MAPK thus promoting oxidative stress and neuroinflammation. Furthermore, also ROCK-1 would be involved in NOX2 activation, therefore it could contribute to the progression of neuronal damage, as well as to the development of AD. In experimental AD models, the accumulation of Aβ activated RAGE, stimulating the NOX2 activation, hence the production of ROS. RAGE would facilitate both the transport of Aβ through the BBB and the pathological accumulation of Aβ in the brain parenchyma. Therefore, the activation of RAGE would induce alterations in BBB permeability and functionality as well as neurodegeneration and cognitive decline. Therefore, treatment with new molecules such as NOX2 and RAGE inhibitors could serve as a basis for the development of promising therapies for pathological conditions associated with NOX2 activation including dementia.

### 6.3. NOX2 in Amyotrophic Lateral Sclerosis

ALS is an adult-onset ND that primarily affects motoneurons. The hallmark of ALS is a progressive degeneration of motoneurons that cause muscle weakness, atrophy, and can also impair both speech and breathing [103]. The progression of ALS is very quick and causes death from respiratory failure within 5 years from the beginning of the illness [104]. The etiology is still unknown, and therefore it is considered a multifactorial disease [105]. Most cases of ALS have sporadic etiology. Nevertheless, 5–10% of ALS is familial, and more than 20 gene mutations have been identified, such as Cu/Zn superoxide dismutase 1 gene (*SOD1*), TAR DNA-binding protein 43 (*TDP43*), fused in sarcoma (*FUS*)/translocated in sarcoma and ubiquitin 2. In ALS patients were found virus particles in several organs and also in CSF. The analyses post-mortem performed have allowed for the detection of viral particles in serum and brain tissue of ALS patients. Therefore, it suggests that viral infections could be implicated in ALS pathology [106]. Certainly, inflammation and oxidative stress play a determinant role in its progression. Indeed, microglia activation induces the release of pro-inflammatory cytokines and ROS, which promote neurodegeneration [105].

The microglial activation and ROS production mediated by NOX2 upregulation it has been identified as an of the main cause of the disease. It was also demonstrated that in ALS the activation of NOX2 is also a transcriptional target of NF-κB in microglia that could be useful for modulating the interaction between oxidative stress and neuroinflammation. In this regard, Zhang et al. showed that in transgenic rats hSOD1-G93A induced i.p. with diphenyl diselenide (50 μmol/kg), a compound known mainly for its antioxidant and anti-inflammatory properties, delayed the progression of the disease and favored the motor neuron survival. Similarly, the authors reported that in primary microglia, the pretreatment with diphenyl diselenide (400 nM) prior to LPS stimulation has downregulated NF-κB signaling involved in the release of inflammatory cytokine and ROS production. Since in microglia NOX2 is the main source of ROS, the pretreatment with diphenyl diselenide has been shown to significantly reduce the expression of Gp91^phox^ subunit of NOX2 and also the intracellular ROS. Moreover, again in microglia, it was shown that diphenyl diselenide pretreatment did not involve the Nfr2 antioxidant pathway (activated following stimulation with LPS) and also confirmed that the reduction of ROS was mediated by NOX2. Additionally, it was shown that diphenyl diselenide blocked the activation of the inflammasome leading also to a reduction of caspase-1 and IL-1β activity [107]. 

The neuroprotective effects and the reduction of neuroinflammation were shown by Boucherie et al. in hSOD1-G93A rats administered intrathecally in the CSF with a single injection of mesenchymal cells 2 × 10^6^ labeled with bromodeoxyuridine. The authors demonstrated that infiltration of mesenchymal cells, as well as their differentiation into astrocytes in the injury site, has reduced the degeneration of motor neurons in the spinal cord, and also prolonged the survival and motor functions of these animals. Moreover, following the mesenchymal cell transplantation, it has been demonstrated a decrease of the inflammation and microglial proliferation, as well as the reduction of NOX2 and COX2. Likewise, it has also been reported that the progression of ALS is related to microglial activation, thus ROS production as well as the NOX2 activation. Therefore, the study results highlighted once again how one reduces excessive inflammation through inhibition of NOX2 and COX2 could be a useful strategy for counteracting the progression of the NDs. In the same way, the measure of changes in the expression of NOX2 could be used as a marker to evaluate the oxidative stress that occurs following microglial activation [108]. 

The passage of microglia from a neuroprotective phenotype M2 to a neurotoxic M1 was shown by Liao et al., in mSOD1-G93A mice. The authors demonstrated that in the mSOD1 microglia of mice in the early stages of ALS an increase in the expression of M2 markers including Ym1, CD163, and Brain-derived neurotrophic factor (BDNF) as well as a lower expression of NOX2 an M1 marker. Conversely, an increase of NOX2 and ROS was demonstrated in the late stages of the disease. Taken together, these results suggested that the disease progression may be characterized by a change of mSOD1 microglia from a neuroprotective to a neurotoxic phenotype. Consequently, NOX2 and its expression levels could be a useful marker for identifying the microglial activation in the different stages of the disease [109]. Both microglial activation and lymphocyte infiltration are processes widely involved in ALS. Zhao et al. showed the interaction between microglia and lymphocytes in mSOD1-G93A mice. Primary microglial cells isolated from the spinal cord of mSOD1 mice (1 × 10^4^ cells/well) were treated with regulatory CD4 + CD25^High^ T lymphocytes (Tregs) or cytotoxic CD4 + CD25− T lymphocytes (Teffs) obtained from spleen and lymph nodes. The authors showed that Tregs (1 × 10^4^ cells/well) co-cultured with mSOD1 microglia of mice, inhibited NOX2 and iNOS through a mechanism mediated by IL-4. The involvement of IL-4 in the modulation of cytotoxicity following microglial activation was demonstrated using the IL-4 blocking antibody, which favored the increase of NOX2 and iNOS levels, as well as the cytotoxicity in the primary microglial cells. Therefore, the expression levels of NOX2 and iNOS following the use of IL-4 blocking antibody were like those shown in the Teffs cells (1 × 10^4^ cells/well) cultured together with the primary microglia SOD1 cells. In the same way, Teffs cells (1 × 10^4^ cells/well) co-cultured with primary microglia SOD1 cells demonstrated that cytotoxicity was suppressed by IL-4, IL-10, and TGF-β. Therefore, this data suggests how Tregs can inhibit Nox-2 and iNOS and thus, cytotoxicity induced following microglial activation [110]. In addition to the involvement of microglial activation, the inflammatory responses that characterize ALS in the different stages of the disease were also shown by Beers et al. in mSOD1 G93A mice. To explain the onset of motor weakness first in the hind limbs and then in the anterior limbs, the authors sacrificed the animals and observed whether T-cell infiltration into the cervical and lumbar spinal cords was related to temporal and functional differences in the inflammatory responses. The results of the study showed a differential response between the protective and inflammatory responses within the cervical and lumbar spinal cords. Indeed, it was shown that the presence of CD4 + T lymphocytes in the lumbar spinal cord region of mSOD1-G93A mice through the increase of anti-inflammatory cytokines such as IL-4 and trophic factors including BDNF, Glial cell line-derived neurotrophic factor (GDNF), reduced the progression of the disease and favored the motor neuron survival. In the same way, the endogenous neuroprotective response of T lymphocytes in the cervical region attenuated the neurotoxic responses of the microglia activation induced by TNF-α, IL-1β, and NOX2. Although there were no differences in the NOX2, TNF-α, and IL-1β levels between the cervical and lumbar spinal cords, the earlier onset of disease symptoms in the hind limbs were attributable to neurotoxic inflammatory responses, thus to Th2 response in the cervical spinal cord and Th1 in the lumbar spinal cord [111]. 

To clarify the role of T cells in ALS, Beers et al., demonstrated in mSOD1-G93A mice the effects of bone marrow (3 × 10^7^ cells per mouse) administered via i.p. Since T lymphocytes and in particular CD4 +, play a neuroprotective role, the authors reported how the loss of the CCR2 receptor involved in the recruitment of T lymphocytes favored the motor neuron degeneration as well as the disease progression. This latter also confirmed by the increase of NOX2 activity, as well as by the reduction of BDNF, GDNF, and glial glutamate transporters. On the contrary, bone marrow transplantation was shown to stimulate the production of T lymphocytes which in turn have suppressed the cytotoxicity and also modulated the microglial activity NOX2 dependent. Therefore, the glial/T-cell interactions could be provided a novel therapeutic target for NDs such as ALS [112].

In ALS mice it was reported that the ROS increase NOX2-mediated following microglial activation can leading to motor neuron death. To investigate how mutant forms of SOD1 can lead to an increase of microglial ROS dependent on NOX2 and thus to neuronal death, Li et al., used Glial MO59J cells or neuronal cells NSC-34 expressing SOD1-G93A. The results of the study performed both in MO59J and NSC-34 expressing SOD1-G93A showed the hyperactivation in the ROS production dependent by Rac1 and NOX2, as well as an increase of toxicity and inflammation, the latter evaluated by the TNFα and NFκB levels. As the mutant forms of SOD1 have a higher affinity for Rac1-GTP, it has been shown that alsin in the presence of SOD1-G93A in glial cells, attenuated the neurotoxicity by reducing Rac1 activation and thus the signaling mediated by NOX2. Therefore, the results obtained suggested how the inflammation and neurotoxicity that characterize ALS involved NOX2 [113].

TDP-43 protein represents a major component of neuronal inclusions in ALS patients. It was found in CSF of ALS patients. To evaluate the effects of TDP-43 in this pathological disease, the latter was administered in the microglia of mice. The first evaluation showed an upregulation of NOX2 mRNA expression and an increase in both TNF-α and IL-1β levels, aimed to examine whether microglia were activated. Instead, co-treatment with PMBS, an LPS inhibitor, provided further confirmation that microglia activation was induced by TDP-43 treatment. This co-treatment did not show significant changes in NOX2 expression and inflammatory cytokines, confirming that microglia activation was not affected by possible LPS contamination during the experiment. Moreover, NLRP3 inflammasome was observed in TDP-43-treated microglia, so it may be responsible for promoting IL-1β transcription as well as caspase-1 activation. In this context, it has been shown the role of CD14 microglia in the activation of the inflammatory process but did not affect the NOX2 expression. Likewise, the TDP-43-treatment in microglia was able to promote the MAPK and NF-κB pathway, triggering the inflammatory process. Interestingly, inflammation could also be affected by AP-1 activation. As evidence AP-1 inhibition induced a decrease in inflammatory factors in microglia TDP-43-treated. For this reason, it was inferred that TDP-43 was able to promote the microglia activation and inflammation causing the motoneurons degeneration over time [114].

Experimental studies have been demonstrated that some compounds such as pro-inflammatory cytokines, ROS, or extracellular ATP could induce neurotoxicity. Indeed, extracellular ATP could stimulate the ionotropic purinergic P2 × 7 receptors promoting an inflammatory response. For this reason, Apolloni et al., conducted an investigation to observe the P2X7 receptor-mediated NOX2 pathway in SOD1-G93A microglia. In this culture, an increase of p67^PHOX^ translocation was observed after treatment with 2′-3′-*O*-(benzoyl-benzoyl) ATP (BzATP) (10–100 μM), a P2X7 receptor agonist suggesting the role of this receptor in inducing activation of the NOX2 pathway. To determine whether P2X7/NOX2 recruited Rac1 into the microglia, the authors infected the G93A microglia with lentivirus or adenovirus showing an increase of CD68 expression, a marker of microglia activated. Furthermore, the translocation of p67^PHOX^ was explored through infection with Rac1 mutant. As evidence, the control was treated with a P2X7 inhibitor demonstrating that NOX2 activation was dependent on the P2X7 receptor. Instead, the p67 ^PHOX^ translocation may depend on Rac1 recruitment. In these cultures, after BzATP treatment, the apocynin was administrated to inhibit NOX2, demonstrating a remarkable decrease in ROS. Hence, the NOX2-P2X7 receptor was involved in the release of oxidized compounds in BzATP-induced microglia. It was observed that stimulation of the P2X7 receptor was able to induce p38 activation. Moreover, ERK 1/2 phosphorylation was more represented in SOD1-G93A. However, ERK1/2 phosphorylation could also be induced by NOX2 activation. Although ROS production was increased by NOX2 activation, it was seen that ERK1/2 activation could participate in their production. Ultimately, it was possible to define a correlation between the P2X7 receptor and ERK1/2 and NOX2 in SOD1-G93A microglia [105].

Instead, Seredenina et al. evaluated the involvement of NOX in the progression of ALS. Therefore, they conducted an experiment in which the expression levels of NOX isoforms in the spinal cord of WT and SOD1-G93A mice were observed at the beginning (90 days) and terminal stage (120 days) of the disease. This observation allowed us to observe an increase of NOX expression levels, especially NOX2 and its subunit (such as p22^PHOX^, p67^PHOX^, p47^PHOX^) related to the progression of ALS. In agreement with this result, the author performed immunostaining in the spinal cord of ALS patients in which NOX2 was more represented. Furthermore, to confirm the involvement of NOX2 in ALS disease, rat RA2 microglia were pretreated with LPS to induce their activation. The findings confirmed an increase in both NOX2 expression and ROS levels. For this reason, treatment with perphenazine or thioridazine showed promising results as inhibitions of the production of NOX2-derived oxidizing compounds in microglia. The same results were found in in vivo experiment in which thioridazine administration can decrease ROS levels in SOD1-G93A mice. Regardless, treatment with perphenazine or thioridazine in SOD1 G93A mice did not increase their survival. Moreover, thioridazine treatment significantly reduced both NOX2 and microglial marker expressions such as Iba1 and CD68. Although thioridazine did not improve disease progression, it had a considerable impact on NOX2 activity [115].

It is widely known that SOD1 mutations represent the cause of neurological diseases such as ALS. In several experimental ALS models, it was observed that mutations in the SOD1 gene are responsible for the overproduction of oxidized compounds (Table 3). Therefore, it has been related that mutated SOD1 can activate Rac1, which consequently activates NOX2 causing the production of harmful ROS. Furthermore, SOD1 mutation in microglial cells would promote neuroinflammation, so affect motor neuron viability. Reduction of ROS release by NOX2 inhibition has already proven to be an effective treatment. Indeed, in microglial cells, apocynin inhibited the translocation of p47^PHOX^ and p67^PHOX^ while perphenazine or thioridazine reduced ROS levels. For this reason, the experimental studies have also targeted the treatment with T lymphocytes showing a progressive increase of anti-inflammatory cytokines such as IL-4 and trophic factors including BDNF, GDNF, slowing the progression of the disease and increasing the survival of the motor neurons. Therefore, further investigations focusing on the inhibition of NOX2 in microglia are needed to slow the course of the disease.

## 7. Conclusions

This manuscript focused on the role of microglial NOX2 involved in neuroinflammation. Indeed, the purpose of this review is to provide a hypothesis on the possible mode of NOX2 activation in microglia triggered following SARS-CoV-2 infection and any neurological repercussions. It is already known that NOX2 activation in microglia amplifies the inflammatory process through the release of oxidized compounds and pro-inflammatory cytokines. To date, there is no clinical treatment capable of counteracting the NOX2 mechanism, however, further inhibition could limit the production of ROS in microglia to attenuate the toxicity in neuronal cells. In this way, it could preventively contribute to counteracting the pathophysiological mechanisms following the SARS-CoV-2 infection. Furthermore, we highlighted the role of NOX2 in both promoting oxidative stress and neuroinflammation with particular relevance for NDs, providing a new insight into the pathophysiology of COVID-19. However, our study has limitations as there are not enough studies that can support the hypothesis of a correlation between COVID-19 and neurodegeneration. Ultimately, it would be interesting to investigate NOX2 activation in microglia following viral infection. Furthermore, NOX2 could be a new useful target for the therapeutic treatment of neurodegenerative NDs such as PD, AD, and ALS.

## Figures and Tables

**Table 1:** Role of NOX2 in Parkinson’s disease.

Models	Treatments	Results	Ref
	In Vitro and Vivo Experimental Models		
Primary microglial cells obtained from whole brains of wild type (WT) or CD11b^−^/^−^ mice; microglia BV2 cell line	α-Syn (50, 100, and 200) nM; pre-treatment with anti-CD11b antibody (2.5 μg/mL) for 30 min.	The translocation of p47^PHOX^ into the membrane and consequently, ROS production in microglia α-Syn-induced. BV2 microglia α-Syn-induced were pre-treated with anti-CD11b antibody to block CD11b activation for demonstrating its implication in NOX2 activation.	[67]
Adult male SD rats (9 weeks old); Primary mesencephalic neuron-glia, microglia-depleted, and mixed-glia cultures; BV2 microglial cell line;	Intraperitoneal injection of HD (400 mg/kg/die) five times in 1 week for consecutive 5 weeks in rats; HD (1, 4, 8, and 16) mM.	Increased levels of both ROS and p47^PHOX^ levels were observed in HD-treated BV2 microglia, demonstrating NOX2 activation. Instead, microglia cells were pre-treated with apocynin (NOX2 inhibitor), further suggesting that NOX2 was responsible for inducing ROS production. Furthermore, integrin αMβ2 (also known as CD11b) has been shown to be involved in NOX2 activation. Indeed, its inhibition would reduce ROS and p47^PHOX^ translocation.	[68]
BV2 microglial cells	Combination of paraquat and maneb (10 + 0.6) μM.	The co-treatment induced an increase in ROS levels and p47^PHOX^ translocation, evidencing NOX2 activation. Furthermore, the inactivation of the C3 receptor (integrins) decreased the production of superoxide and translocation of p47^PHOX^, supporting the involvement of integrins in the activation of NOX2. The inactivation of integrins impacted the Src-ERK pathway.	[69]
Male C57BL/6J (NOX2^+^/^+^ and NOX2^−^/^−^) mice	Intraperitoneal injection of paraquat + maneb (10 + 30) mg/kg 2 times in one week for 6 weeks in mice; Pre-treatment with Taurine (150 mg/kg)	Exposure to paraquat + maneb induced both an increase in NOX2 subunits (p47^PHOX^ and gp91^PHOX^), as well as α-Syn expression levels which demonstrate neurodegeneration. Moreover, NOX2 could induce microglial polarization. Instead, pretreatment with taurine counteracted neurodegeneration and reduced translocation of NOX2 subunits.	[70]
Male C57BL/6J (NOX2^+^/^+^ and NOX2^−^/^−^) mice; neuron-glia culture cells obtained from mouse or rat embryos; HAPI cells	Injection of A29-V40 (α-Syn) peptide (5 mg/kg) for 24 h in mice; Treatment with PMA (100 Nm) or LPS (150 EU/mL); Pre-treatment with apocynin (0.25 mM)	PMA and A29-V40 treatment-induced translocation of p47^PHOX^ and p67^PHOX^ and could bind gp91^PHOX^ subunit in mice. The co-treatment with apocynin restored the viability of dopaminergic neurons and their ability to DA uptake. Instead, microglia culture pre-treated with H_2_O_2_ in gp91^PHOX −^/^−^ microglia promoted the phosphorylation of p47^PHOX^ and Erk1/2 demonstrating that several factors could affect NOX2 activation.	[71]
Primary midbrain neuron-glia cultures obtained from brains of SD rats; primary glia cultures obtained from NOX2^+^/^+^ and NOX2^−^/^−^ mice; Male C57BL/6J (NOX2^+^/^+^ and NOX2^−^/^−^) mice;	Cultures were treated with LPS (0.5 ng/mL) and/or Syn (20 nmol/L) for 8 days LPS (0.2 ug/uL) + syn (0.0125 ug/uL) treatment was administered in mice. Pre-treatment with DPI (0.01 and 0.1) μM	ROS levels, p47^PHOX,^ and gp91^PHOX^ were increased after LPS + syn treatment. Interestingly, a decrease in both dopaminergic levels and DA uptake was found in NOX2^+^/^+^ mice. Conversely, both the number of microglia and the ROS levels increased. The same results were found in rat primary midbrain neuron-glia cultures obtained from NOX2+/+ and NOX2^−^/^−^ mice. Instead, DPI improved the viability of dopaminergic neurons and reduced ROS.	[72]
neuron-glia mixed culture cells of Time-pregnant Fisher F344 rats Wild-type C57BL/6J (gp91*^phox^*^+^/^+^) and NOX2-deficient (gp91*^phox^*^−^/^−^) mice	LPS (15 ng/mL) or MPP^+^ (0.25 μM) treatment MPTP injections (20 mg/kg, s.c.) for 6 days. One day prior to MPTP injection, clozapine or CNO (1 mg/kg, s.c.) was administered twice daily for 21 consecutive days and pre-treatment with DPI (3 mg/kg, i.p)	LPS-treatment in NOX2^+^/^+^ mice promoted both mRNA NOX2 expression and release of ROS in a time-dependent manner. Co-treatment with CNO or NDC showed how NOX2 activation influenced DA uptake and release of pro-inflammatory compounds. DPI-Pre-treatment reduced both ROS and inflammatory cytokines such as TNFα, MCP-1, and LPS-induced IL-1β	[73]
B6.129S6-*Cybb^tm1Din^* (NOX2^−^/^−^) and C57BL/6J 000,664 (NOX2^+^/^+^) mice	Intraperitoneal injection of LPS (5 mg/kg).	LPS-treatment in C57BL/6J mice promoted both mRNA NOX2 expression and release of ROS in a time-dependent manner. Instead, pre-treatment with DPI reduced both ROS and inflammatory cytokines such as TNFα, MCP-1and IL-1β demonstrating that NOX2 promoting microglia activation, subsequently the release of inflammatory cytokines.	[74]
Mixed-glia cultures of B10.129P2(B6)-IL-10^tm1Cgn^/J (IL-10 knockout or IL-10^−^/^−^) mice and their WT or IL-10^+^/^+^ control mice (C57BL/10J), as well as B6N.129S2-Casp1^tm1Flv^/J (caspase-1 knockout or CASP-1^−^/^−^) mice and their WT (CASP-1^+^/^+^) control mice.	Intranigral injection of LPS (3 μg)	NOX2 activation and the consequent increase in ROS were responsible for the activation of NLPR3 inflammasome. The increased levels of IL-10 were able to suppress ROS-NOX2 induced and to block the NLPR3 activation, preventing neuroinflammation.	[75]
Microglial cultures of male C57BL/6J and NOD2 knockout (NOD2^−^/^−^) mice. BV2 microglial cells SH-SY5Y cells	Injection of 6-OHDA (2 μL) Injection of MDP (4 μg/μL) in the right striatum	Treatment with MDP or 6-OHDA induced a reduction in DA and an increase in both apoptotic proteins and inflammatory cytokines. Instead, increased NOX2, NOD2, and iNOS promoting neuroinflammation was observed in 6-OHDA-induced microglia.	[76]
Ten week-old male C57BL/6 (gp91^PHOX−^/^−^) and WT mice Microglial cultures C57BL/6) and gp91^PHOX−^/^−^ mice	6-OHDA (10 µg/µL) was unilaterally injected into the right striatum Minocycline (40 mg/kg) was injected(i.p) 7 days before PD induction	Treatment with 6-OHDA in the striatum of gp91^PHOX−^/^−^ mice reduced the dopaminergic neurons, explaining NOX2-mediated neurotoxicity. Furthermore, co-treatment with minocycline promoted the neurodegeneration and release of TNFα in gp91^PHOX+^/^+^ mice, supporting NOX2 activation.	[77]
Microglial cultures of C57 BL/6J (NOX2^+^/^+^ and NOX2^−^/^−^) mice.	Fe^2+^-treatment (5, 25, and 100) μM	Treatment with Fe^2+^ significantly increases both p47^PHOX^ and gp91^PHOX^ expression, suggesting Fe2^+^-induced NOX2 activation. Moreover, an increase in mRNA expression and protein levels of p38, ERK 1/2, and JNK was observed, therefore Fe^2+^ exposure could promote neuroinflammation.	[78]
Mesencephalic neuron-glia, microglia-depleted, and microglia-enriched cultures of C57BL/6J, SP-deficient (*TAC1*^−^/^−^), and SP receptor-deficient (NK1R^−^/^−^)mice	Treatment with LPS (15 × 106 EU/kg) or MPTP (15 mg/kg) for 6 days	Significant loss of dopaminergic neurons was observed in WT mice treated with SP + LPS or SP + MPP + compared to gp91^PHOX−^/^−^ culture, thus it was inferred that NOX2 may play a role in promoting neurotoxicity. Moreover, it was observed an increase in the translocation of p47^PHOX^ and p67^PHOX^ as well as of several inflammatory factors such as TNFα, iNOS, and MCP-1, suggesting activation of NOX2. Furthermore, MAPK and NF-Κb pathways were activated by NOX2 in microglia after toxicity-induced.	[79]
Mesencephalic neuron-glia cultures from the ventral mesencephalon of embryonic Fischer 334 rats and also on A53T mutant α-synuclein transgenic mice.	The intranigral and intraperitoneal injection of LPS (5 mg/kg) and subcutaneous injection MPTP (15 mg/kg)	Increased levels in both gp91^PHOX^ and G6PD were observed after LPS or MPTP treatment in mice. Neuron-glia culture treated with LPS demonstrated an increase of NADPH levels and G6PD activity, so it could be the promoter of NOX2 activation that induces neuroinflammation. Moreover, an increase of both G6PD and NOX2 in microglia are responsible to implement oxidative stress, the NF-Kb translocation, and subsequent neurodegeneration.	[63]

WT: wild type; CD11b: cluster of differentiation molecule 11b; α-Syn: α-Synuclein; ROS: reactive oxygen species; NOX2: NADPH oxidase 2; SD: Sprague Dawley; HD: 2,5-hexanedione; Src: Proto-oncogene tyrosine-protein kinase; ERK: extracellular signal-regulated kinase; DA: dopamine; HAPI: highly aggressively proliferating immortalized; PMA: phorbol 12-myristate 13-acetate; LPS: lipopolysaccharide; H_2_O_2_: hydrogen peroxide; DPI: diphenyleneiodonium; MPP^+^: 1-methyl-4-phenylpyridinium; MPTP: 1-methyl-4-phenyl-1,2,3,6-tetrahydropyridine; CNO: clozapine N-oxide; NDC: N-Desmethylclozapine; TNFα: tumor necrosis factor-α; MCP-1: monocyte chemoattractant protein-1; IL-1β: interleukin-1beta; IL-10: interleukin-10; KO: knockdown; casp-1: caspase-1; NLRP3: NLR family pyrin domain containing 3; 6-OHDA: 6-hyroxydopamine; MDP: muramyl dipeptide; NOD2: Nucleotide-binding oligomerization domain-containing protein; Fe^2+^: iron (II); p38: p38 mitogen-activated protein kinase; JNK: c-Jun N-terminal kinase; SP: substance P; iNOS: inducible nitric oxidase synthase; MAPK: mitogen-activated protein kinase; NF-Kb: nuclear factor-Κb; G6PD: glucose-6-phosphatase-dehydrogenase.

**Table 2:** Role of NOX2 in Alzheimer’s disease.

Models	Treatments	Results	Ref.
	In Vitro and Vivo Experimental Models		
BV-2 mouse microglial cells, mesencephalic samples of WT C57BL/6 mice, NOX2-KO mice, and post-mortem mesencephalic human samples	Aβ_1–42_ (0.1–10 µM), apocynin (20 μM) or NOX2tat (10 μM)	In vitro, treatment with apocynin or NOX2tat has significantly reduced ROS production. While a reduction of ROS and IL-1β and inhibition of ERK1/2 signaling was observed in NOX2-KO mice compared to both mesencephalic samples of mice in elderly WT mice and mesencephalic samples of elderly humans.	[7]
Human neuroblast oma cells	Fasudil (1 µM) approximately	Compound 3 reduced inflammation as demonstrated by the reduction of pro-inflammatory mediators including IL-6, IL-1β, and TNFα.	[99]
R1.40 transgenic mice, human THP-1 monocytes	animal food ibuprofen enriched in a final dosage (62.5 mg/kg), Aβ_25–35_ (60 μM)	The treatment reduced Aβ aggregation. Moreover, in primary murine microglia, the pretreatment with ibuprofen reduced the superoxide production Aβ-induced. In the same way, in ibuprofen-treated human THP-1 monocytes, by blocking Vav phosphorylation Aβ-induced, inhibited NOX2 and ROS production.	[100]
hCMEC/D3 cells	Aβ_1–42_ (1 μM), DPI (10 nM and 100 nM), allopurinol (10 nM and 100 nM)	The pretreatment with allopurinol and DPI before inducing hCMEC/D3 with Aβ led to the reduction of ROS levels and restored the levels of occludin and claudin. Moreover, the blocking of RAGE and NOX2 by anti-RAGE blocking antibody prevented the cytotoxicity induced by Aβ and thus its transport through the BBB in the brain	[101]
RAW264.7 mouse macrophage cell line and the BV2 mouse microglial cells	LPS or ATP (1 mM or 5 mM) and apocynin (30 nmol).	Treatment with CXCL1 led to an increase in both ROS and NOX2. Moreover, apocynin pre-treatment inhibited both NOX2 and CXCL1, involved in promoting the proliferation of neural cells in the presence of ROS.	[102]

WT: wild type; KO: knockdown; Aβ_1–__42:_ beta-amyloid_1–42__;_ NOX2: NADPH oxidase2; ROS: reactive oxygen species; IL-1β: interleukin-1beta; ERK: extracellular signal-regulated kinase; IL-6: interleukin-6; TNFα: tumor necrosis factor-α; Aβ_25–35_: beta-amyloid_25–35_; DPI: diphenyleneiodonium; RAGE: Receptor for advanced glycation end products; BBB: blood brain barrier; LPS: lipopolysaccharide; ATP: denosine 5′-triphosphate; CXCL1: C-X-C motif chemokine ligand 1.

**Table 3:** Role of NOX2 in Amyotrophic Lateral Sclerosis.

Models	Treatments	Results	Ref
	In Vitro and Vivo Experimental Models		
hSOD1-G93A rats and primary microglia	diphenyl diselenide (50 μmol/kg) via intraperitoneal diphenyl diselenide (400 nM)	In vivo, diphenyl diselenide reduced the progression of the disease and favored motor neuron survival. While the same treatment in microglia has reduced the expression of the gp91^PHOX^ subunit of NOX2 and also the production of ROS. Moreover, diphenyl diselenide blocked the activation of the inflammasome and reduced the levels of caspase-1 and IL-1β.	[107]
hSOD1-G93A rats	a single injection of mesenchymal cells 2 × 10^6^ labeled via intrathecal in the CSF	The infiltration and differentiation of mesenchymal cells in the injury site reduced the neurodegeneration of motor neurons in the spinal cord. Moreover, the transplantation of mesenchymal cells reduced inflammation and microglial activation as well as NOX2 and COX2 levels.	[108]
mSOD1 microglia	mSOD1 microglia (2000 cells/well) co-culturing with motoneurons	The study has demonstrated changes in mSOD1 microglia of the mouse from a neuroprotective to a neurotoxic phenotype, as well as increased in the expression of NOX2, ROS, and markers including Ym1, CD163, and BDNF.	[109]
mSOD1-G93A mice	mSOD1 microglia (1 × 10^4^ cells/well) co-cultured with Tregs (1 × 10^4^ cells/well) or Teffs cells (1 × 10^4^ cells/well)	The study has shown the interaction between microglial activation and T lymphocytes, through a mechanism that involved IL-4 in the modulation of cytotoxicity. Indeed, Tregs through IL-4 reduced NOX2 and iNOS levels in primary microglial cells. Similarly, IL-4 inhibition promoted the increase of NOX2 and iNOS.	[110]
mSOD1-G93A mice	analysis of spinal cord sections	CD4 ^+^ infiltration in the lumbar spinal cord increased IL-4, BDNF, and GDNF levels, as well as promoted motor neuron survival. Similarly, it was shown in the cervical spinal cord both a reduction of the microglial activation and also a reduction in TNF-α, IL-1β, and NOX2.	[111]
mSOD1-G93A mice	bone marrow (3 × 10^7^ cells per mouse) via intraperitoneal	The bone marrow transplantation has led to the recruitment of CD4^+^ lymphocytes which have preserved motor neurons from the neurodegeneration. Consequently, it was demonstrated the reduction of NOX2 levels and also the increase of BDNF, GDNF, and glutamate transporters.	[112]
MO59J glial cells and NSC-34 neuronal cells and SOD1-G93A mice	Glial MO59J cells (1.0 × 10^6^) and NSC-34 (0.5 × 10^6^) infected with adenoviruses (1000 particles per cell)	Both MO59J glial cells and NSC-34 neuronal cells SOD1G93A-expressing have demonstrated an increase in Rac1, NOX2, ROS, TNFα, and NF-κB levels. Similarly, alsin has shown a higher affinity for Rac1-GTP in MO59J cells, thus reducing Rac1 activation and therefore NOX2 activity.	[113]
Microglial cells and primary motoneuron of spinal cords obtained from C57BL/6 mice	TDP-43 treatment (500 ng/mL) in microglia for 2 days; LPS (40 ng/mL) and PMBS (4 μg/mL)	TDP-43 induced an increase in NOX2 expression and TNF-α and IL-1β levels as well as activation of NLRP3 inflammasome. Similarly, TDP-43 treatment in microglia was able to promote the MAPK and NF-κB pathway. Instead, PMBS co-treatment confirmed that microglia activation depended on TDP-43 and showed no significant changes in NOX2 expression and inflammatory cytokines.	[114]
SOD1-G93A transgenic mice	microglia SOD1-G93A treated with BzATP (10–100 μM)	BzATP improved the NOX2 activity and consequently ROS production through a mechanism mediated by translocation of p67^PHOX^. Moreover, the administration of apocyanine in microglia treated with BzATP has inhibited NOX2 and reduced ROS. Similarly, a relationship between NOX2 and ERK1/2 phosphorylation mediated by P2 × 7 receptors was demonstrated.	[105]
SOD1-G93A mice and Ra2 microglia	Ra2 microglia (10,000 cells/well) LPS-treated (5 µg/mL), perphenazine (3 mg/kg) or thioridazine (10 mg/kg) intraperitoneal administration	In vivo, it was shown an increase in the expression levels of p22^PHOX^, p67^PHOX^, and p47^PHOX^ (NOX2 subunits). Moreover, thioridazine reduced NOX2 and ROS levels, as well as the expression levels of microglial markers Iba1 and CD68. Besides, either treatment with perphenazine or thioridazine in SOD1 G93A mice did not increase motor neuron survival, while in microglia inhibited ROS production and NOX2 activity.	[115]

NOX2: NADPH oxidase2; ROS: reactive oxygen species; IL-1β: interleukin-1beta; CSF: cerebrospinal fluid; SOD1: Superoxide dismutase; COX2: cyclooxygenase2; CD163: Cluster of Differentiation 163; BDNF: Brain-derived neurotrophic factor; IL-4: interleukin-4; iNOS: inducible nitric oxidase synthase; GDNF: Glial cell-derived neurotrophic factor; TNFα: tumor necrosis factor-α; Rac1: Ras-related C3 botulinum toxin substrate 1; NF-κB: nuclear factor kappa-light-chain-enhancer of activated B cells; GTP: Guanosine-5′-triphosphate; TDP-43: TAR DNA-binding protein 43; NLRP3: NLR family pyrin domain containing 3; LPS: lipopolysaccharide; PMBS: polymyxin B sulfate; MAPK: mitogen-activated protein kinase; BzATP:2′-3′-*O*-(benzoyl-benzoyl) ATP; ERK: extracellular signal-regulated kinase; Iba1: ionized calcium-binding adapter molecule 1; CD68: Cluster of Differentiation 68.

## Data Availability

Not applicable.

## References

[1] Battaglini D., Brunetti I., Anania P., Fiaschi P., Zona G., Ball L., Giacobbe D.R., Vena A., Bassetti M., Patroniti N. (2020). Neurological Manifestations of Severe SARS-CoV-2 Infection: Potential Mechanisms and Implications of Individualized Mechanical Ventilation Settings. Front. Neurol..

[2] Meyerowitz E.A., Richterman A., Gandhi R.T., Sax P.E. (2021). Transmission of SARS-CoV-2: A Review of Viral, Host, and Environmental Factors. Ann. Intern. Med..

[3] Cavalli E., Bramanti A., Ciurleo R., Tchorbanov A.I., Giordano A., Fagone P., Belizna C., Bramanti P., Shoenfeld Y., Nicoletti F. (2020). Entangling COVID-19 associated thrombosis into a secondary antiphospholipid antibody syndrome: Diagnostic and therapeutic perspectives (Review). Int. J. Mol. Med..

[4] Gavriatopoulou M., Korompoki E., Fotiou D., Ntanasis-Stathopoulos I., Psaltopoulou T., Kastritis E., Terpos E., Dimopoulos M.A. (2020). Organ-specific manifestations of COVID-19 infection. Clin. Exp. Med..

[5] Liu T., Zhang L., Joo D., Sun S.-C. (2017). NF-κB signaling in inflammation. Signal Transduct. Target. Ther..

[6] De Andrade E.G., Šimončičová E., Carrier M., Vecchiarelli H.A., Robert M.È., Tremblay M.-È. (2021). Microglia Fighting for Neurological and Mental Health: On the Central Nervous System Frontline of COVID-19 Pandemic. Front. Cell. Neurosci..

[7] Geng L., Fan L.M., Liu F., Smith C., Li J.-M. (2020). Nox2 dependent redox-regulation of microglial response to amyloid-β stimulation and microgliosis in aging. Sci. Rep..

[8] Kumar A., Barrett J., Alvarez-Croda D.-M., Stoica B.A., Faden A.I., Loane D.J. (2016). NOX2 drives M1-like microglial/macrophage activation and neurodegeneration following experimental traumatic brain injury. Brain Behav. Immun..

[9] Fan L.M., Geng L., Cahill-Smith S., Liu F., Douglas G., McKenzie C.-A., Smith C., Brooks G., Channon K.M., Li J.-M. (2019). Nox2 contributes to age-related oxidative damage to neurons and the cerebral vasculature. J. Clin. Investig..

[10] Cahill-Smith S., Li J.-M. (2014). Oxidative stress, redox signalling and endothelial dysfunction in ageing-related neurodegenerative diseases: A role of NADPH oxidase. Br. J. Clin. Pharmacol..

[11] Loffredo L., Ettorre E., Zicari A.M., Inghilleri M., Nocella C., Perri L., Spalice A., Fossati C., De Lucia M.C., Pigozzi F. (2020). Oxidative Stress and Gut-Derived Lipopolysaccharides in Neurodegenerative Disease: Role of NOX2. Oxidative Med. Cell. Longev..

[12] Violi F., Oliva A., Cangemi R., Ceccarelli G., Pignatelli P., Carnevale R., Cammisotto V., Lichtner M., Alessandri F., De Angelis M. (2020). Nox2 activation in Covid-19. Redox Biol..

[13] Li Y., Zhou W., Yang L., You R. (2020). Physiological and pathological regulation of ACE2, the SARS-CoV-2 receptor. Pharmacol. Res..

[14] Hamming I., Timens W., Bulthuis M.L., Lely A.T., Navis G., van Goor H. (2004). Tissue distribution of ACE2 protein, the functional receptor for SARS coronavirus. A first step in understanding SARS pathogenesis. J. Pathol..

[15] Doobay M.F., Talman L.S., Obr T.D., Tian X., Davisson R.L., Lazartigues E. (2007). Differential expression of neuronal ACE2 in transgenic mice with overexpression of the brain renin-angiotensin system. Am. J. Physiol. Integr. Comp. Physiol..

[16] Shang J., Wan Y., Luo C., Ye G., Geng Q., Auerbach A., Li F. (2020). Cell entry mechanisms of SARS-CoV-2. Proc. Natl. Acad. Sci. USA.

[17] Sulzer D., Antonini A., Leta V., Nordvig A., Smeyne R.J., Goldman J.E., Al-Dalahmah O., Zecca L., Sette A., Bubacco L. (2020). COVID-19 and possible links with Parkinson’s disease and parkinsonism: From bench to bedside. NPJ Park. Dis..

[18] Uhlén M., Fagerberg L., Hallström B.M., Lindskog C., Oksvold P., Mardinoglu A., Sivertsson Å., Kampf C., Sjöstedt E., Asplund A. (2015). Tissue-based map of the human proteome. Science.

[19] Gupta A., Madhavan M.V., Sehgal K., Nair N., Mahajan S., Sehrawat T.S., Bikdeli B., Ahluwalia N., Ausiello J.C., Wan E.Y. (2020). Extrapulmonary manifestations of COVID-19. Nat. Med..

[20] Butowt R., Bilinska K. (2020). SARS-CoV-2: Olfaction, Brain Infection, and the Urgent Need for Clinical Samples Allowing Earlier Virus Detection. ACS Chem. Neurosci..

[21] Baig A.M., Khaleeq A., Ali U., Syeda H. (2020). Evidence of the COVID-19 Virus Targeting the CNS: Tissue Distribution, Host–Virus Interaction, and Proposed Neurotropic Mechanisms. ACS Chem. Neurosci..

[22] Wang H.-Y., Li X.-L., Yan Z.-R., Sun X.-P., Han J., Zhang B.-W. (2020). Potential neurological symptoms of COVID-19. Ther. Adv. Neurol. Disord..

[23] Desforges M., Le Coupanec A., Stodola J.K., Meessen-Pinard M., Talbot P.J. (2014). Human coronaviruses: Viral and cellular factors involved in neuroinvasiveness and neuropathogenesis. Virus Res..

[24] Zhou Z., Kang H., Li S., Zhao X. (2020). Understanding the neurotropic characteristics of SARS-CoV-2: From neurological manifestations of COVID-19 to potential neurotropic mechanisms. J. Neurol..

[25] Dube M., Le Coupanec A., Wong A.H.M., Rini J.M., Desforges M., Talbot P.J. (2018). Axonal Transport Enables Neuron-to-Neuron Propagation of Human Coronavirus OC43. J. Virol..

[26] Román G.C., Spencer P.S., Reis J., Buguet A., Faris M.E.A., Katrak S.M., Láinez M., Medina M.T., Meshram C., Mizusawa H. (2020). The neurology of COVID-19 revisited: A proposal from the Environmental Neurology Specialty Group of the World Federation of Neurology to implement international neurological registries. J. Neurol. Sci..

[27] Davies J., Randeva H.S., Chatha K., Hall M., Spandidos D.A., Karteris E., Kyrou I. (2020). Neuropilin-1 as a new potential SARS-CoV-2 infection mediator implicated in the neurologic features and central nervous system involvement of COVID-19. Mol. Med. Rep..

[28] Filho A.J.M.C., Gonçalves F., Mottin M., Andrade C.H., Fonseca S.N.S., Macedo D.S. (2021). Repurposing of Tetracyclines for COVID-19 Neurological and Neuropsychiatric Manifestations: A Valid Option to Control SARS-CoV-2-Associated Neuroinflammation?. J. Neuroimmune Pharmacol..

[29] Jakhmola S., Indari O., Chatterjee S., Jha H.C. (2020). SARS-CoV-2, an Underestimated Pathogen of the Nervous System. SN Compr. Clin. Med..

[30] Matschke J., Lütgehetmann M., Hagel C., Sperhake J.P., Schröder A.S., Edler C., Mushumba H., Fitzek A., Allweiss L., Dandri M. (2020). Neuropathology of patients with COVID-19 in Germany: A post-mortem case series. Lancet Neurol..

[31] Deigendesch N., Sironi L., Kutza M., Wischnewski S., Fuchs V., Hench J., Frank A., Nienhold R., Mertz K.D., Cathomas G. (2020). Correlates of critical illness-related encephalopathy predominate postmortem COVID-19 neuropathology. Acta Neuropathol..

[32] Bhat S.A., Goel R., Shukla S., Shukla R., Hanif K. (2017). Angiotensin Receptor Blockade by Inhibiting Glial Activation Promotes Hippocampal Neurogenesis Via Activation of Wnt/β-Catenin Signaling in Hypertension. Mol. Neurobiol..

[33] Rana I., Suphapimol V., Jerome J.R., Talia D.M., Deliyanti D., Wilkinson-Berka J.L. (2020). Angiotensin II and aldosterone activate retinal microglia. Exp. Eye Res..

[34] Cui C., Xu P., Li G., Qiao Y., Han W., Geng C., Liao D., Yang M., Chen D., Jiang P. (2019). Vitamin D receptor activation regulates microglia polarization and oxidative stress in spontaneously hypertensive rats and angiotensin II-exposed microglial cells: Role of renin-angiotensin system. Redox Biol..

[35] Labandeira-Garcia J.L., Rodríguez-Perez A.I., Garrido-Gil P., Rodriguez-Pallares J., Lanciego J.L., Guerra M.J. (2017). Brain Renin-Angiotensin System and Microglial Polarization: Implications for Aging and Neurodegeneration. Front. Aging Neurosci..

[36] Rodriguez-Perez A.I., Borrajo A., Rodriguez-Pallares J., Guerra M.J., Labandeira-Garcia J.L. (2015). Interaction between NADPH-oxidase and Rho-kinase in angiotensin II-induced microglial activation. Glia.

[37] Borrajo A., Rodriguez-Perez A.I., Diaz-Ruiz C., Guerra M.J., Labandeira-Garcia J.L. (2014). Microglial TNF-α mediates enhancement of dopaminergic degeneration by brain angiotensin. Glia.

[38] Garrido-Gil P., Rodriguez-Pallares J., Meijide A.D., Guerra M.J., Labandeira-Garcia J.L. (2013). Brain angiotensin regulates iron homeostasis in dopaminergic neurons and microglial cells. Exp. Neurol..

[39] Bhat S.A., Sood A., Shukla R., Hanif K. (2019). AT2R Activation Prevents Microglia Pro-inflammatory Activation in a NOX-Dependent Manner: Inhibition of PKC Activation and p47phox Phosphorylation by PP2A. Mol. Neurobiol..

[40] Kumar D., Jahan S., Khan A., Siddiqui A.J., Redhu N.S., Wahajuddin, Khan J., Banwas S., Alshehri B., Alaidarous M. (2021). Neurological Manifestation of SARS-CoV-2 Induced Inflammation and Possible Therapeutic Strategies Against COVID-19. Mol. Neurobiol..

[41] Mishra R., Banerjea A.C. (2021). SARS-CoV-2 Spike Targets USP33-IRF9 Axis via Exosomal miR-148a to Activate Human Microglia. Front. Immunol..

[42] Hosp J.A., Dressing A., Blazhenets G., Bormann T., Rau A., Schwabenland M., Thurow J., Wagner D., Waller C., Niesen W.D. (2021). Cognitive impairment and altered cerebral glucose metabolism in the subacute stage of COVID-19. Brain J. Neurol..

[43] Bedard K., Krause K.H. (2007). The NOX Family of ROS-Generating NADPH Oxidases: Physiology and Pathophysiology. Physiol. Rev..

[44] Ha E.-M., Oh C.-T., Bae Y.S., Lee W.-J. (2005). A Direct Role for Dual Oxidase inDrosophilaGut Immunity. Science.

[45] Altenhöfer S., Radermacher K.A., Kleikers P.W.M., Wingler K., Schmidt H.H.H.W. (2015). Evolution of NADPH Oxidase Inhibitors: Selectivity and Mechanisms for Target Engagement. Antioxid. Redox Signal..

[46] Segal A.W., García R., Goldstone H., Cross A.R., Jones O.T. (1981). Cytochrome b-245 of neutrophils is also present in human monocytes, macrophages and eosinophils. Biochem. J..

[47] Elsen S., Doussiere J., Villiers C.L., Faure M., Berthier R., Papaioannou A., Grandvaux N., Marche P.N., Vignais P.V. (2004). Cryptic O2—Generating NADPH oxidase in dendritic cells. J. Cell Sci..

[48] Jesaitis A.J., Buescher E.S., Harrison D., Quinn M.T., Parkos C.A., Livesey S., Linner J. (1990). Ultrastructural localization of cytochrome b in the membranes of resting and phagocytosing human granulocytes. J. Clin. Investig..

[49] Chanock S.J., Faust L.R., Barrett D., Bizal C., Maly F.E., Newburger P.E., Ruedi J.M., Smith R.M., Babior B.M. (1992). O2—Production by B lymphocytes lacking the respiratory burst oxidase subunit p47phox after transfection with an expression vector containing a p47phox cDNA. Proc. Natl. Acad. Sci. USA.

[50] Huang J., Hitt N.D., Kleinberg M.E. (1995). Stoichiometry of p22-phox and gp91-phox in phagocyte cytochrome b558. Biochemistry.

[51] Weiss S.J., Klein R., Slivka A., Wei M. (1982). Chlorination of Taurine by Human Neutrophils. J. Clin. Investig..

[52] Clark R.A., Volpp B.D., Leidal K.G., Nauseef W.M. (1990). Two cytosolic components of the human neutrophil respiratory burst oxidase translocate to the plasma membrane during cell activation. J. Clin. Investig..

[53] Bäumer A.T., Freyhaus H.T., Sauer H., Wartenberg M., Kappert K., Schnabel P., Konkol C., Hescheler J., Vantler M., Rosenkranz S. (2008). Phosphatidylinositol 3-Kinase-dependent Membrane Recruitment of Rac-1 and p47phox Is Critical for α-Platelet-derived Growth Factor Receptor-induced Production of Reactive Oxygen Species. J. Biol. Chem..

[54] Cheng G., Lambeth J.D. (2004). NOXO1, Regulation of Lipid Binding, Localization, and Activation of Nox1 by the Phox Homology (PX) Domain. J. Biol. Chem..

[55] Koga H., Terasawa H., Nunoi H., Takeshige K., Inagaki F., Sumimoto H. (1999). Tetratricopeptide Repeat (TPR) Motifs of p67 Participate in Interaction with the Small GTPase Rac and Activation of the Phagocyte NADPH Oxidase. J. Biol. Chem..

[56] Lavigne M.C., Malech H.L., Holland S.M., Leto T.L. (2000). Genetic requirement of p47 phox for superoxide production by murine microglia. FASEB J..

[57] Lapouge K., Smith S.J.M., Groemping Y., Rittinger K. (2002). Architecture of the p40-p47-p67 Complex in the Resting State of the NADPH Oxidase. J. Biol. Chem..

[58] Massenet C., Chenavas S., Cohen-Addad C., Dagher M.-C., Brandolin G., Pebay-Peyroula E., Fieschi F. (2005). Effects of p47phox C terminus phosphorylations on binding interactions with p40phox and p67phox. Structural and functional comparison of p40phox and p67phox SH3 domains. J. Biol. Chem..

[59] Singel K.L., Segal B.H. (2016). NOX2-dependent regulation of inflammation. Clin. Sci..

[60] Tarafdar A., Pula G. (2018). The Role of NADPH Oxidases and Oxidative Stress in Neurodegenerative Disorders. Int. J. Mol. Sci..

[61] Diebold B.A., Smith S.M., Li Y., Lambeth J.D. (2015). NOX2 as a Target for Drug Development: Indications, Possible Complications, and Progress. Antioxid. Redox Signal..

[62] Ali K., Morris H.R. (2014). Parkinson’s disease: Chameleons and mimics. Pr. Neurol..

[63] Tu D., Gao Y., Yang R., Guan T., Hong J.-S., Gao H.-M. (2019). The pentose phosphate pathway regulates chronic neuroinflammation and dopaminergic neurodegeneration. J. Neuroinflamm..

[64] Zhang P.-L., Chen Y., Zhang C.-H., Wang Y.-X., Fernandez-Funez P. (2017). Genetics of Parkinson’s disease and related disorders. J. Med Genet..

[65] Hayes M.T. (2019). Parkinson’s Disease and Parkinsonism. Am. J. Med..

[66] Cannon J.R., Greenamyre J.T. (2013). Gene–Environment interactions in Parkinson’s disease: Specific evidence in humans and mammalian models. Neurobiol. Dis..

[67] Hou L., Bao X., Zang C., Yang H., Sun F., Che Y., Wu X., Li S., Zhang D., Wang Q. (2018). Integrin CD11b mediates α-synuclein-induced activation of NADPH oxidase through a Rho-dependent pathway. Redox Biol..

[68] Zhang C., Hou L., Yang J., Che Y., Sun F., Li H., Wang Q. (2018). 2,5-Hexanedione induces dopaminergic neurodegeneration through integrin αMβ2/NADPH oxidase axis-mediated microglial activation. Cell Death Dis..

[69] Hou L., Wang K., Zhang C., Sun F., Che Y., Zhao X., Zhang D., Li H., Wang Q. (2018). Complement receptor 3 mediates NADPH oxidase activation and dopaminergic neurodegeneration through a Src-Erk-dependent pathway. Redox Biol..

[70] Che Y., Hou L., Sun F., Zhang C., Liu X., Piao F., Zhang D., Li H., Wang Q. (2018). Taurine protects dopaminergic neurons in a mouse Parkinson’s disease model through inhibition of microglial M1 polarization. Cell Death Dis..

[71] Wang S., Chu C.-H., Guo M., Jiang L., Nie H., Zhang W., Wilson B., Yang L., Stewart T., Hong J.-S. (2016). Identification of a specific α-synuclein peptide (α-Syn 29-40) capable of eliciting microglial superoxide production to damage dopaminergic neurons. J. Neuroinflamm..

[72] Zhang W., Gao J.-H., Yan Z.-F., Huang X.-Y., Guo P., Sun L., Liu Z., Hu Y., Zuo L.-J., Yu S.-Y. (2016). Minimally Toxic Dose of Lipopolysaccharide and α-Synuclein Oligomer Elicit Synergistic Dopaminergic Neurodegeneration: Role and Mechanism of Microglial NOX2 Activation. Mol. Neurobiol..

[73] Jiang L., Wu X., Wang S., Chen S.-H., Zhou H., Wilson B., Jin C., Lu R.-B., Xie K., Wang Q. (2016). Clozapine metabolites protect dopaminergic neurons through inhibition of microglial NADPH oxidase. J. Neuroinflamm..

[74] Qin L., Liu Y., Hong J.-S., Crews F.T. (2013). NADPH oxidase and aging drive microglial activation, oxidative stress, and dopaminergic neurodegeneration following systemic LPS administration. Glia.

[75] Gao Y., Tu D., Yang R., Chu C.-H., Hong J.-S., Gao H.-M. (2020). Through Reducing ROS Production, IL-10 Suppresses Caspase-1-Dependent IL-1β Maturation, thereby Preventing Chronic Neuroinflammation and Neurodegeneration. Int. J. Mol. Sci..

[76] Cheng L., Chen L., Wei X., Wang Y., Ren Z., Zeng S., Zhang X., Wen H., Gao C., Liu H. (2018). NOD2 promotes dopaminergic degeneration regulated by NADPH oxidase 2 in 6-hydroxydopamine model of Parkinson’s disease. J. Neuroinflamm..

[77] Hernandes M.S., Santos G.D.R., Café-Mendes C.C., Lima L.S., Scavone C., Munhoz C., Britto L.R.G. (2013). Microglial Cells Are Involved in the Susceptibility of NADPH Oxidase Knockout Mice to 6-Hydroxy-Dopamine-Induced Neurodegeneration. PLoS ONE.

[78] Zhang W., Yan Z.-F., Gao J.-H., Sun L., Huang X.-Y., Liu Z., Yu S.-Y., Cao C.-J., Zuo L.-J., Chen Z.-J. (2014). Role and Mechanism of Microglial Activation in Iron-Induced Selective and Progressive Dopaminergic Neurodegeneration. Mol. Neurobiol..

[79] Wang Q., Chu C.-H., Qian L., Chen S.-H., Wilson B., Oyarzabal E., Jiang L., Ali S., Robinson B., Kim H.-C. (2014). Substance P Exacerbates Dopaminergic Neurodegeneration through Neurokinin-1 Receptor-Independent Activation of Microglial NADPH Oxidase. J. Neurosci..

[80] Gandy S. (2005). The role of cerebral amyloid beta accumulation in common forms of Alzheimer disease. J. Clin. Investig..

[81] Ecanobbio I., Abubaker A.A., Evisconte C., Etorti M., Epula G. (2015). Role of amyloid peptides in vascular dysfunction and platelet dysregulation in Alzheimer’s disease. Front. Cell. Neurosci..

[82] Bloom G.S. (2014). Amyloid-β and Tau. JAMA Neurol..

[83] Simpson D.S.A., Oliver P.L. (2020). ROS Generation in Microglia: Understanding Oxidative Stress and Inflammation in Neurodegenerative Disease. Antioxidants.

[84] Von Bernhardi R., Eugenín-von Bernhardi L., Eugenín J. (2015). Microglial cell dysregulation in brain aging and neurodegeneration. Front. Aging Neurosci..

[85] Chen S., Luo D., Streit W.J., Harrison J.K. (2002). TGF-β1 upregulates CX3CR1 expression and inhibits fractalkine-stimulated signaling in rat microglia. J. Neuroimmunol..

[86] Herrera-Molina R., Flores B., Orellana J., Von Bernhardi R. (2012). Modulation of interferon-γ-induced glial cell activation by transforming growth factor β1: A role for STAT1 and MAPK pathways. J. Neurochem..

[87] Weiss A., Attisano L. (2013). The TGFbeta Superfamily Signaling Pathway. Wiley Interdiscip. Rev. Dev. Boil..

[88] Nakanishi H., Wu Z. (2009). Microglia-aging: Roles of microglial lysosome- and mitochondria-derived reactive oxygen species in brain aging. Behav. Brain Res..

[89] Siniachenko O.V., Diadyk A.I., Bagriĭ E.A. (1991). Neurological aspects of microcrystalline arthropathies. Sov. Meditsina.

[90] Blau C.W., Cowley T.R., O’Sullivan J., Grehan B., Browne T.C., Kelly L., Birch A., Murphy N., Kelly A.M., Kerskens C.M. (2012). The age-related deficit in LTP is associated with changes in perfusion and blood-brain barrier permeability. Neurobiol. Aging.

[91] Ii M., Sunamoto M., Ohnishi K., Ichimori Y. (1996). β-Amyloid protein-dependent nitric oxide production from microglial cells and neurotoxicity. Brain Res..

[92] Qin L., Liu Y., Cooper C., Liu B., Wilson B., Hong J.-S. (2002). Microglia enhance β-amyloid peptide-induced toxicity in cortical and mesencephalic neurons by producing reactive oxygen species. J. Neurochem..

[93] Combs C.K., Johnson D.E., Karlo J.C., Cannady S.B., Landreth G.E. (2000). Inflammatory mechanisms in Alzheimer’s disease: Inhibition of beta-amyloid-stimulated proinflammatory responses and neurotoxicity by PPARgamma agonists. J. Neurosci. Off. J. Soc. Neurosci..

[94] Griffin W., Sheng J., Royston M., Gentleman S., McKenzie J., Graham D., Roberts G., Mrak R. (2006). Glial-Neuronal Interactions in Alzheimer’s Disease: The Potential Role of a ‘Cytokine Cycle’ in Disease Progression. Brain Pathol..

[95] Reed-Geaghan E.G., Savage J.C., Hise A.G., Landreth G.E. (2009). CD14 and Toll-Like Receptors 2 and 4 Are Required for Fibrillar A -Stimulated Microglial Activation. J. Neurosci..

[96] Wilkinson B.L., Landreth G.E. (2006). The microglial NADPH oxidase complex as a source of oxidative stress in Alzheimer’s disease. J. Neuroinflamm..

[97] Fragoso-Morales L., Correa-Basurto J., Rosales-Hernández M. (2021). Implication of Nicotinamide Adenine Dinucleotide Phosphate (NADPH) Oxidase and Its Inhibitors in Alzheimer’s Disease Murine Models. Antioxidants.

[98] Miyano K., Ueno N., Takeya R., Sumimoto H. (2006). Direct Involvement of the Small GTPase Rac in Activation of the Superoxide-producing NADPH Oxidase Nox1. J. Biol. Chem..

[99] Alokam R., Singhal S., Srivathsav G.S., Garigipati S., Puppala S., Sriram D., Perumal Y. (2014). Design of dual inhibitors of ROCK-I and NOX2 as potential leads for the treatment of neuroinflammation associated with various neurological diseases including autism spectrum disorder. Mol. BioSyst..

[100] Wilkinson B.L., Cramer P.E., Varvel N., Reed-Geaghan E., Jiang Q., Szabo A., Herrup K., Lamb B.T., Landreth G.E. (2012). Ibuprofen attenuates oxidative damage through NOX2 inhibition in Alzheimer’s disease. Neurobiol. Aging.

[101] Carrano A., Hoozemans J.J., Van Der Vies S.M., Rozemuller A.J., Van Horssen J., De Vries H.E. (2011). Amyloid Beta Induces Oxidative Stress-Mediated Blood–Brain Barrier Changes in Capillary Amyloid Angiopathy. Antioxid. Redox Signal..

[102] Shang Y., Tian L., Chen T., Liu X., Zhang J., Liu D., Wei J., Fang W., Chen Y., Shang D. (2019). CXCL1 promotes the proliferation of neural stem cells by stimulating the generation of reactive oxygen species in APP/PS1 mice. Biochem. Biophys. Res. Commun..

[103] Hardiman O., Al-Chalabi A., Chio A., Corr E.M., Logroscino G., Robberecht W., Shaw P.J., Simmons Z., Berg L.H.V.D. (2017). Amyotrophic lateral sclerosis. Nat. Rev. Dis. Prim..

[104] Yamanaka K., Komine O. (2018). The multi-dimensional roles of astrocytes in ALS. Neurosci. Res..

[105] Apolloni S., Parisi C., Pesaresi M.G., Rossi S., Carrì M.T., Cozzolino M., Volonté C., D’Ambrosi N. (2013). The NADPH Oxidase Pathway Is Dysregulated by the P 2 × 7 Receptor in the SOD1-G93A Microglia Model of Amyotrophic Lateral Sclerosis. J. Immunol..

[106] Bonafede R., Mariotti R. (2017). ALS Pathogenesis and Therapeutic Approaches: The Role of Mesenchymal Stem Cells and Extracellular Vesicles. Front. Cell. Neurosci..

[107] Zhang C., Wang H., Liang W., Yang Y., Cong C., Wang Y., Wang S., Wang X., Wang D., Huo D. (2021). Diphenyl diselenide protects motor neurons through inhibition of microglia-mediated inflammatory injury in amyotrophic lateral sclerosis. Pharmacol. Res..

[108] Boucherie C., Schäfer S., Lavand’Homme P., Maloteaux J.-M., Hermans E. (2009). Chimerization of astroglial population in the lumbar spinal cord after mesenchymal stem cell transplantation prolongs survival in a rat model of amyotrophic lateral sclerosis. J. Neurosci. Res..

[109] Liao B., Zhao W., Beers D.R., Henkel J.S., Appel S.H. (2012). Transformation from a neuroprotective to a neurotoxic microglial phenotype in a mouse model of ALS. Exp. Neurol..

[110] Zhao W., Beers D.R., Liao B., Henkel J.S., Appel S.H. (2012). Regulatory T lymphocytes from ALS mice suppress microglia and effector T lymphocytes through different cytokine-mediated mechanisms. Neurobiol. Dis..

[111] Beers D.R., Zhao W., Liao B., Kano O., Wang J., Huang A., Appel S.H., Henkel J.S. (2011). Neuroinflammation modulates distinct regional and temporal clinical responses in ALS mice. Brain Behav. Immun..

[112] Beers D.R., Henkel J.S., Zhao W., Wang J., Appel S.H. (2008). CD4+ T cells support glial neuroprotection, slow disease progression, and modify glial morphology in an animal model of inherited ALS. Proc. Natl. Acad. Sci. USA.

[113] Li Q., Spencer N.Y., Pantazis N.J., Engelhardt J.F. (2011). Alsin and SOD1G93A Proteins Regulate Endosomal Reactive Oxygen Species Production by Glial Cells and Proinflammatory Pathways Responsible for Neurotoxicity. J. Biol. Chem..

[114] Zhao W., Beers D.R., Bell S., Wang J., Wen S., Baloh R.H., Appel S.H. (2015). TDP-43 activates microglia through NF-κB and NLRP3 inflammasome. Exp. Neurol..

[115] Seredenina T., Nayernia Z., Sorce S., Maghzal G.J., Filippova A., Ling S.-C., Basset O., Plastre O., Daali Y., Rushing E.J. (2016). Evaluation of NADPH oxidases as drug targets in a mouse model of familial amyotrophic lateral sclerosis. Free. Radic. Biol. Med..

